# Understanding pathways from implementation to sustainment: a longitudinal, mixed methods analysis of promising practices implemented in the Veterans Health Administration

**DOI:** 10.1186/s13012-024-01361-z

**Published:** 2024-05-07

**Authors:** Andrea L. Nevedal, Marilla A. Opra Widerquist, Caitlin M. Reardon, Maria Arasim, George L. Jackson, Brandolyn White, Madison Burns, Gemmae M. Fix, Kathryn DeLaughter, Sarah L. Cutrona, Allen L. Gifford, Guneet K. Jasuja, Timothy P. Hogan, Heather A. King, Blake Henderson, Laura J. Damschroder

**Affiliations:** 1 Center for Clinical Management Research (CCMR), Veterans Affairs Ann Arbor Healthcare System, 2215 Fuller Rd, Mail Stop 152, Ann Arbor, MI 48105 USA; 2 Center of Innovation to Accelerate Discovery and Practice Transformation (ADAPT), Durham Veterans Affairs Health Care System, Durham, NC USA; 3 Peter O’Donnell Jr. School of Public Health, University of Texas Southwestern Medical Center, Dallas, TX USA; 4 Center for Healthcare Organization & Implementation Research (CHOIR), Bedford & Boston Veterans Affairs Medical Centers, Bedford & Boston, MA USA; 5 Section of General Internal Medicine, Boston University Chobanian & Avedisian School of Medicine, Boston, MA USA; 6 Department of Population and Quantitative Health Sciences, University of Massachusetts Chan Medical School, Worcester, MA USA; 7 Division of General Internal Medicine, University of Massachusetts Chan Medical School, Worcester, MA USA; 8 Department of Health Law, Policy & Management, Boston University School of Public Health, Boston, MA USA; 9 Department of Population Health Sciences, Duke University, Durham, NC USA; 10 Division of General Internal Medicine, Duke University, Durham, NC USA; 11 Innovation Ecosystem, United States Veterans Health Administration, Washington, DC, USA

**Keywords:** Implementation outcomes, Implementation, Sustainment outcomes, Sustainability, Sustainment, Maintenance, Longitudinal, Survey, Sustainment determinants, Consolidated Framework for Implementation Research (CFIR), Qualitative Methods, Mixed Methods

## Abstract

**Background:**

The Veterans Health Administration (VHA) is the United States largest learning health system. The Diffusion of Excellence (DoE) program is a large-scale model of diffusion that identifies and diffuses evidence-informed practices across VHA. During the period of 2016-2021, 57 evidence-informed practices were implemented across 82 VHA facilities. This setting provides a unique opportunity to understand sustainment determinants and pathways. Our objective was to characterize the longitudinal pathways of practices as they transition from initial implementation to long-term sustainment at each facility.

**Methods:**

A longitudinal, mixed-methods evaluation of 82 VHA facilities. Eighty-two facility representatives, chosen by leadership as points-of-contact for 57 DoE practices, were eligible for post-implementation interviews and annual sustainment surveys. Primary outcomes (implementation, sustainment), and secondary outcomes (institutionalization, effectiveness, anticipated sustainment) at four time-points were collected. We performed descriptive statistics and directed content analysis using Hailemariam et al.’s factors influencing sustainment.

**Results:**

After approximately five years post-implementation (e.g., 2021 sustainment outcomes), of the 82 facilities, about one-third fully sustained their practice compared to one-third that did not fully sustain their practice because it was in a “liminal” stage (neither sustained nor discontinued) or permanently discontinued. The remaining one-third of facilities had missing 2021 sustainment outcomes. A higher percentage of facilities (70%) had inconsistent primary outcomes (changing over time) compared to facilities (30%) with consistent primary outcomes (same over time). Thirty-four percent of facilities with sustained practices reported resilience since they overcame implementation and sustainment barriers. Facilities with sustained practices reported more positive secondary outcomes compared to those that did not sustain their practice. Key factors facilitating practice sustainment included: demonstrating practice effectiveness/benefit, sufficient organizational leadership, sufficient workforce, and adaptation/alignment with local context. Key factors hindering practice sustainment included: insufficient workforce, not able to maintain practice fidelity/integrity, critical incidents related to the COVID-19 pandemic, organizational leadership did not support sustainment of practice, and no ongoing support.

**Conclusions:**

We identified diverse pathways from implementation to sustainment, and our data underscore that initial implementation outcomes may not determine long-term sustainment outcomes. This longitudinal evaluation contributes to understanding impacts of the DoE program, including return on investment, achieving learning health system goals, and insights into achieving high-quality healthcare in VHA.

**Supplementary Information:**

The online version contains supplementary material available at 10.1186/s13012-024-01361-z.

Contributions to the literature

- Contrary to literature, more facilities had practices with inconsistent (changing) primary outcomes from initial implementation to long-term sustainment. Initial success/failure did not always predict future success/failure.
- Future research should explore if facilities with practices in a “liminal” stage of sustainment (neither sustained nor discontinued) benefit from additional support/intervention.
- Common barriers to long-term sustainment included: insufficient *workforce*, *not able to maintain practice fidelity/integrity*, critical incidents related to the COVID-19 pandemic, *organizational leadership did not support sustainment of practice,* and *no ongoing support.*
- Common facilitators to long-term sustainment included: demonstrating practice *effectiveness/benefit*, sufficient *organizational leadership*, sufficient *workforce*, and *adaptation/alignment* with local context.

## Background

It is well known that it takes years to implement evidence-informed practices (EIP) in real world settings [1]. However, understanding if EIPs are not only implemented, but sustained over a longer period is less frequently reported. Sustainment is defined as the extent to which an EIP is (or is not) in use after an initial implementation phase and is measured by assessing outcomes [2, 3]. Aarons et al. describe the need to study sustainment as “critical,” and “at least as important as the study of implementation,” given that over half of implemented innovations are not sustained over the long-term with fidelity [4]. Sustainment is important because otherwise “time and fiscal investments in implementation are wasted and public health impact is limited [4].”

Important facets for enhancing our knowledge about sustainment and developing more systematic and effective approaches to measuring sustainment include: 1) differentiating sustainment versus initial implementation and defining when it begins [5, 6]; 2) studying sustainment longitudinally [5–7]; 3) describing and critiquing frameworks employed [5], 4) studying use of sustainment strategies, fidelity checklists, and adaptations [5, 8]; 5) describing factors contributing to long-term sustainment success or failure; and 6) using pragmatic approaches to assessing sustainment [7, 9].

Though systematically studying EIP sustainment over the long-term is “critical,” it is complex and often difficult for researchers to attempt due to limited resources and fixed funding cycles [9], participant burden [10], and methodological challenges [9]. Given these challenges, EIP sustainment is reported less often [6, 7], there is no gold standard way for studying sustainment, and there is an increased need for implementation scientists to develop pragmatic ways to study the dynamic nature of long-term sustainment [6]. With the exception of the Provider Report of Sustainment Scale (PReSS) [11], few pragmatic sustainment measures exist, and even application of the PReSS in understanding sustainment across diverse EIPs and settings has not been evaluated.

As part of the Veterans Health Administration (VHA)’s learning health system goals, the Diffusion of Excellence (DoE) was created as a novel nationwide program to support broad EIP diffusion. Our team has evaluated implementation and sustainment of DoE EIPs since its inception [3, 12–20]. Although there is no gold standard definition, we considered initial sustainment as approximately one year after the 6-9-months of facilitated implementation support period and considered longer-term sustainment beginning approximately two years later after the implementation period. From 2016 to 2021, the DoE has supported implementation and diffusion of 57 diverse EIPs known as “Promising Practices” (hereafter: practice) across 82 facilities in the VHA. There were 82 VHA employees, chosen by leadership, to be the facility representative since they were the key point-of-contact and responsible for implementing one of the 57 practices. In some cases, more than one facility implemented the same practice (i.e., 19/57 practices were implemented at more than one facility). Although facilities received approximately 6-9-months of facilitated implementation support for their practice, their implementation and initial sustainment outcomes varied widely [14]. Previously [14], we found initial implementation status did not necessarily predict initial sustainment. For example, some facilities had an initial unsuccessful implementation outcome, but facility efforts continued and went on to achieve full implementation and initial sustainment of their practices at follow-up. In contrast to published literature [8, 21], these results differ from long-standing ideas that initial implementation success predicts future sustainment success [14]. Most literature on sustainment actually focuses on sustainability (anticipated/predictions of future sustainment) [3] or on initial sustainment following successful initial implementation [8], leaving understandings of delayed implementers behind. Even less attention is paid to understanding longer-term sustainment outcomes [5, 7].

The aim of this evaluation was to extend existing sustainment knowledge by reporting sustainment outcomes and characterizing longitudinal patterns from initial implementation to long-term sustainment of DoE’s diverse practices at each facility. In addition, we describe lessons learned and offer future directions for evaluating longer-term sustainment.

## Methods

### Setting

The VHA DoE is one of few large-scale models of diffusion. The DoE seeks to identify, support, and disseminate EIPs across VHA, which is comprised of more than 1,200 facilities. The DoE sponsors annual “Shark Tank” competitions, in which regional and facility leaders bid on the opportunity to implement a practice, coupled with 6-9 months of facilitated implementation support. For additional detail on DoE, see previous publications [3, 13–20, 22] and Additional file 1. Practices are eligible for submission to DoE Shark Tank competitions if they are supported by evidence from research studies and/or administrative or clinical experience and have been implemented in at least one facility. In addition, DoE practices must address VHA patient, employee, and/or facility priorities. Over 2,000 diverse practices were submitted for consideration between Shark Tank Cohorts 1–5. The DoE designated 57 of these as “Promising Practices” and these were adopted at 82 facilities (19 practices were adopted by more than one facility). Two additional practices were implemented outside of standard DoE processes; these are not included in this evaluation. As part of an ongoing DoE evaluation, we conducted interviews and administered surveys to facility representatives, responsible for implementing their practice at a VHA facility, to elicit information about implementation and sustainment of EIPs in local settings (Additional file 1. Further DoE Description).

Per regulations outlined in Veterans Health Administration Program Guide 1200.21, the evaluation of DoE has been designated a non-research quality improvement activity [23].

### Sample

The unit of analysis for this evaluation was the facility (*N*=82) where 57 different practices were implemented from Cohorts 1-5 [24]. These facilities were spread across all 21 VHA geographic regions. Using purposeful criterion sampling, 82 facility representatives were deemed eligible since they were chosen by leadership as the point-of-contact for the practice [24]. Facility representatives encompassed diverse roles across VHA (e.g., nurse, pharmacist, housekeeping aid, engineer, medical assistant) because of the wide range of practices that were implemented. Facility representatives were usually from departments where the practice was implemented/sustained (e.g., environmental services supervisor for a practice addressing housekeeping) or they were in a systems redesign or innovation specialist role that could speak to all ongoing innovations. See Additional file 1 for a description of DoE processes and each practice as well as a map showing practice spread across VHA geographic regions.

### Longitudinal dataset

The dataset for Cohorts 1-5 spanned from 2016 to 2021 and included one semi-structured interview completed by facility representatives approximately 1-2 months after implementation (Cohorts 1-4) and up to three subsequent sustainment surveys administered in 2019, 2020, and 2021 (Cohorts 1-5). Depending on the timing of Cohorts, the 82 facility representatives completed up to 4 timepoints (see Table 1). Due to shifting evaluation priorities, Cohort 5 received the implementation outcome question via survey rather than semi-structured interview.

**Table 1:** Cohort year and data points

**Cohorts**	**Implementation Outcome Year**^a^	**Sustainment Outcome 2019**	**Sustainment Outcome 2020**	**Sustainment Outcome 2021**	**Total Timepoints**
Cohort 1	2016	Yes	Yes	Yes	4
Cohort 2	2017	Yes	Yes	Yes	4
Cohort 3	2018	Yes	Yes	Yes	4
Cohort 4	2019	N/A	Yes	Yes	3
Cohort 5	2020	N/A	N/A	Yes	2

^a^Implementation follow-up was only provided in sustainment surveys for facilities that did not fully implement their practice or were missing data

### Recruitment

Facility representatives were invited via email to participate in a semi-structured interview and REDCap surveys (Additional file 2). To enhance recruitment, we completed three follow-ups via email or instant messaging [25]. We asked participants who indicated they were no longer involved in their practice to provide us with another contact. Facility representatives were excluded from future surveys if they noted that their practice was permanently discontinued or if they did not respond to three consecutive timepoints.

### DOE evaluation and outcomes

#### Evaluation

Our primary evaluation question was: What are the longitudinal pathways from implementation to sustainment of DoE’s Promising Practices at each facility? See Table 2 for outcomes and definitions, questions, and response options.

**Table 2:** Interview/survey questions to assess outcomes and timepoints

**Primary Measure**	**Definition**	**Primary Outcome Measures**	**Timing of Data Collection**
**Practice Implementation**^a^	Extent to which practice’s core components and activities were initially implemented	Question: Was this practice implemented? Response: fully implemented, partially implemented, not implemented Why is your practice [status]?:	2016, 2017, 2018, 2019, 2020
**Practice Sustainment**^b^	Extent to which practice’s core components and activities were in use after initial implementation	Question: What is the current status of this practice? Response: practice is in use/in place, practice is partially in use/in place, practice is temporarily not in use/in place, practice has been discontinued permanently Why is your practice [current status]?:	2019, 2020, 2021
**Secondary Measure**	**Definition**	**Secondary Outcome Measure**	
**Practice Institutionalization**^b^	Extent to which practice’s core components and activities were part of routine care and work processes [27, 28]	Question: Is this practice considered routine, usual practice when it is place? (i.e., practice is nearly always used or done when appropriate by all individuals involved) Response: yes, partially, no Please explain:	2021
**Practice Effectiveness**^b^	Extent to which practice was demonstrating effectiveness	Question: Is the practice demonstrating effectiveness when it is in use? Response: yes, partially, no Please explain:	2021
**Sustainability**^b^^,c^	Likelihood practice’s core components and activities will be in use in the future	Question: What is the likelihood this practice will be sustained in the future: Response: very unlikely, unlikely, neither likely/unlikely, likely, very likely Please explain what will make sustainment harder: Please explain what will make sustainment easier:	2021

^a^Cohorts 1-4 were given the practice implementation measure via semi-structured interviews. Cohort 5 was given this item via survey

^b^Measures were given via survey to Cohorts 1-5

^c^Sustainability is an anticipated outcome [2]

Primary implementation outcome

Implementation outcomes (Table 2) were based on facility representatives’ responses to qualitative and quantitative data obtained from in-depth interviews and sustainment surveys. Implementation outcomes were classified into the following: fully implemented (core elements of the practice were implemented as intended), partially implemented (some but not all core elements of the practice were implemented as intended), not implemented (core elements of the practice were not implemented as intended) or missing (facility representative did not provide a response).

Primary and secondary sustainment outcomes

Sustainment outcomes (Table 2) involved 4 multiple-choice survey questions, which were based on published literature [26–28]; we assessed current practice sustainment, sustainability (anticipated outcome), institutionalization, and effectiveness. Open-ended text boxes were provided after each question, so facility representatives could contextualize their responses (i.e., provide a rationale for their current/anticipated outcome and explain barriers/facilitators to sustainment).

We included 9 Likert Scale determinant questions with follow-up text boxes to understand factors influencing sustainment (e.g., practice priority) [26–28]. However, these data were excluded due to both the low number and inadequate responses to text boxes, which made it unclear which ratings represented relevant barriers and/or facilitators.

Based on responses to open-ended text boxes during our prior evaluation phases [14], we updated the 2021 survey to include a *temporarily not sustained* response option. The *temporarily not sustained* category aimed to capture a more nuanced understanding of sustainment, which included practices that would usually be in place but were currently paused due to, e.g., the COVID-19 pandemic, loss of staff.

## Analysis

Outcome data obtained from 2016-2021 were compiled into a Microsoft Excel matrix to facilitate within and across facility analysis and comparison of each practice pathway from implementation to sustainment (e.g., implementation, implementation follow-up if not initially successful, sustainment 2019, sustainment 2020, sustainment 2021). We used Microsoft Excel to calculate descriptive statistics, response rates, summarize outcomes, and organize longitudinal pathways. We then categorized implementation to sustainment outcome pathways into the following two patterns: 1) consistent outcomes because the outcomes were the same over time (e.g., implemented and then consistently sustained, not implemented and then consistently not sustained, consistently missing) and 2) inconsistent outcomes because outcomes changed over time (e.g., implemented and then not sustained, not implemented and then implemented and sustained, or had a missing outcome). In addition, we categorized practices into the following types: 1) clinical interventions addressing health care priorities, 2) process improvements addressing work related challenges; and 3) staff interventions addressing employee priorities.

We performed directed content analysis of optional open-ended text boxes containing brief responses (approximately 1-2 sentences) [29] using Hailemariam et al.’s [5] systematic review, which is organized by Wiltsey-Stirman et al.’s influences on sustainability framework [8]. These factors are noted in italics in the results. New sustainment factors were created as needed using the updated Consolidated Framework for Implementation Research (CFIR) (e.g., critical incidents) [30] and other sustainment literature (e.g., sustained attention to topic/priority) [27].

## Results

Eighty-two facilities from DoE Cohorts 1-5 collectively implemented 57 diverse practices. Twenty-five out of 57 practices (44%) were clinical interventions addressing a wide range of health care priorities, such as wound care, oral care, and medication safety. Twenty-three out of 57 practices (40%) were process improvements addressing diverse work related challenges (e.g., automated billing for home oxygen, SharePoint tool for construction safety, workflow management for artificial limbs). Nine out of 57 practices (16%) were staff interventions addressing various employee priorities, such as nursing stay interviews, new hire welcome program, and women’s health education. Fifty-three percent of practices (30/57) had a virtual component. Additional file 1 describes each of the 57 practices, which covered a wide range of VHA priorities around patient, staff, and health system needs.

As of 2021 (timepoints ranged from 1-5 years after the 6-9-month facilitated implementation support period), about one-third of facility representatives reported their practice was fully sustained, one-third reported their practice was not fully sustained, and one-third did not respond. One facility representative was missing outcomes for all time-points. Thirty percent (25/82) of facility representatives reported consistent (the same) outcomes from initial implementation to sustainment and 70% (57/82) of facility representatives reported inconsistent (changing) outcomes from implementation to sustainment.

**Fig. 1:**
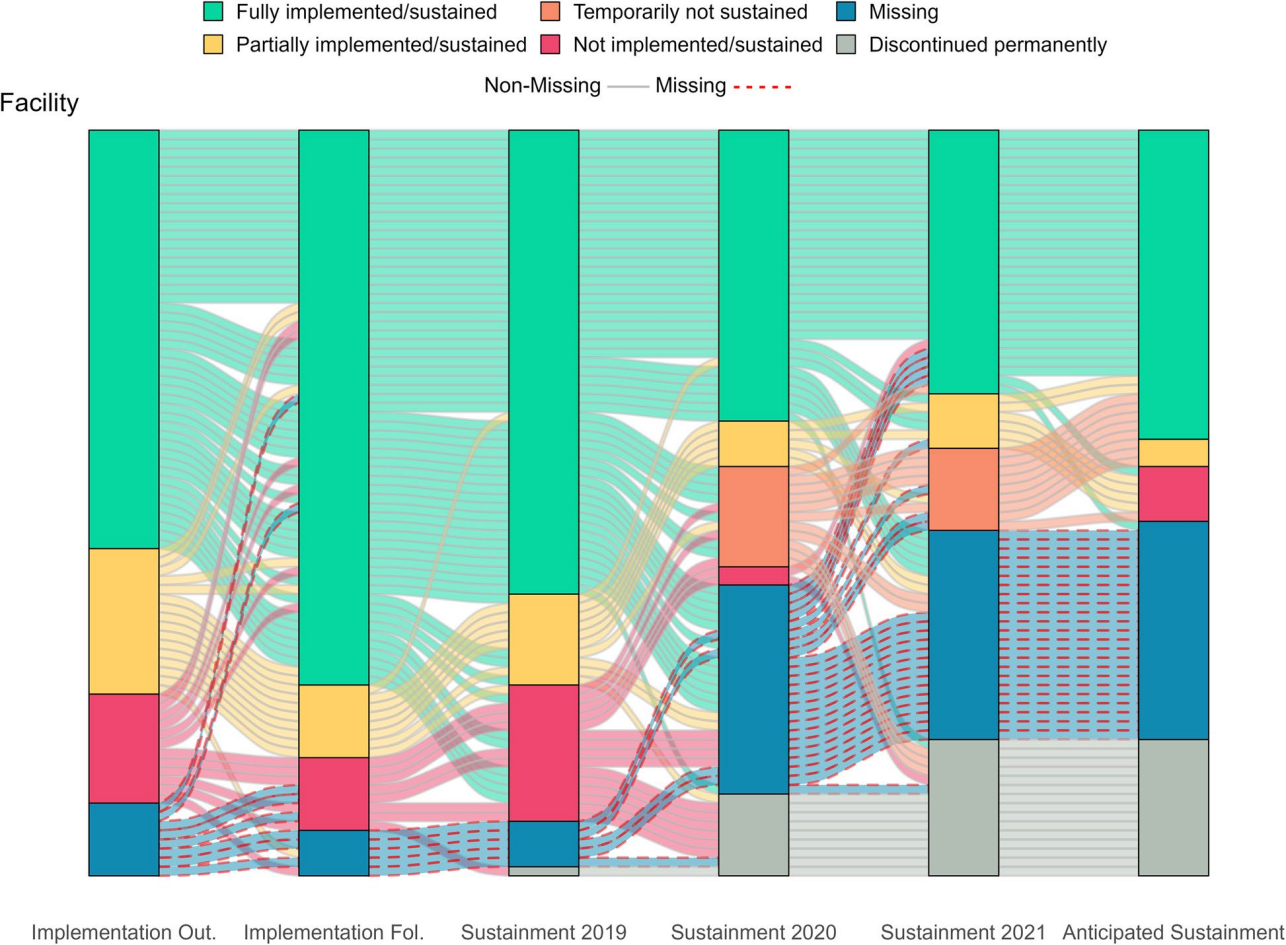
Pathways for all facilities. *Implementation follow-up was only provided for facilities that did not fully implement their practice or were missing data. **Cohort 4 practices only have sustainment outcomes for 2020 and 2021. Cohort 5 practices only have sustainment outcomes for 2021. For the purposes of the figure visual, their statuses were carried over from earlier timepoints

**Fig. 2:**
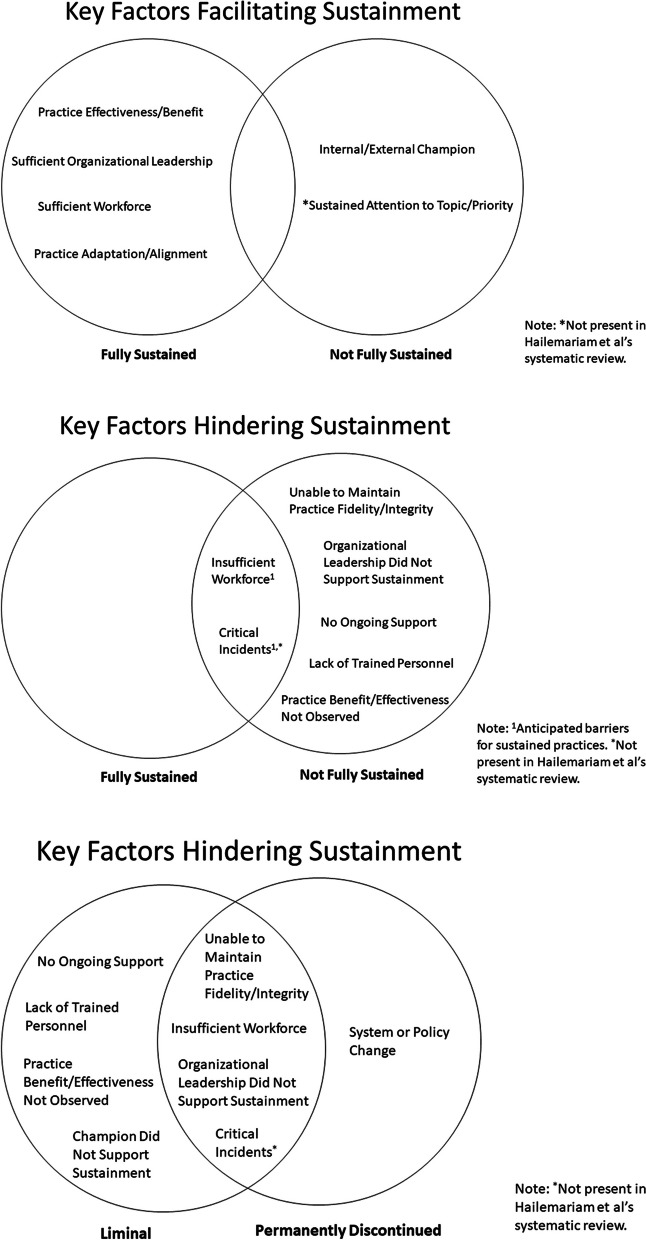
Venn diagram comparing factors that facilitate and hinder sustainment

Figure 1 visually displays all longitudinal pathways, showing shifts in practice implementation/sustainment status over time. The main facilitating and hindering factors influencing sustainment, identified from directed content analysis of open-ended text boxes, are noted in italics below. Additionally, Fig. 2 visually compares these key factors that facilitate and hinder sustainment by outcomes. Tables 3 and 4 list all factors that facilitate and hinder sustainment for the 66% (33/59) of facility representatives who responded to optional open-ended text boxes. Exemplar quotes are provided in Tables 3 and 4 and Figs. 4, 5, 7 and 8. Additional file 3 provides more details, showing sustainment outcomes separately by practice type.

**Table 3:** Factors that facilitate sustainment

**Hailemariam et al.'s** **Facilitating Factors**	**Wiltsey Stirman et al.’s Framework**	**Fully Sustained**	**Not fully sustained**	**Example quotation**
EIP effectiveness or benefit	Innovation characteristics	10	1	“The [EIP] has been successful and continues to guide our practice”
Organizational leadership	Context	8	1	“We have support from leadership and the department”
Workforce	Capacity	6	0	“The primary team is doing well to manage the process with little oversight and facilitator involvement”
Adaptation/Alignment	Processes & interactions	5	0	“[EIP] was adapted to accommodate more telehealth and fewer face to face encounters during COVID 19”
Ongoing support	Processes & interactions	4	1	“Our facility offers strong support for the [EIP] which has allowed us to expand beyond our own facility”
EIP fit	Innovation characteristics	2	1	“It also fits within the mission of [top VHA initiative]”
Critical incidents^a^	Outer Setting^a^	2	0	“Virtual technology helped keep practice ongoing during COVID”
Ability to maintain EIP fidelity/integrity	Innovation characteristics	1	1	“[EIP] is used on a daily basis”
Integration of rules & policies	Processes & interactions	1	0	“[EIP] made an expectation for all nurses and providers”
Resources	Capacity	1	0	“Equipment is not an issue”
Community stakeholder support/involvement	Capacity	1	1	“I met with stakeholders outside the VA in an effort to get equipment and support for our Veterans in these areas”
Internal/external EIP champions	Capacity	0	4	“Unit champion ensures practice is continued”
Training & education	Processes & interactions	0	1	“Previous work with [EIP staff] trainings have facilitated the early success of the pilot program”
Sustained attention to topic/priority^a^	N/A^a^	0	2	“We still use some of the [EIP elements] we set in place during the process”
Total^b^		41	13	

^a^Not present in Hailemariam et al.'s factors

^b^Totals are based on 66% (39/59) of facility representatives who provided brief, open-ended text box responses in the 2021 survey. Some responders had provided more than one type of barrier

**Table 4:** Factors that hinder sustainment

**Hailemariam et al.’s** **Hindering Factors**	**Wiltsey Stirman et al.’s Framework**	**Fully Sustained**	**Not fully sustained**	**Example quotation**
Workforce	Capacity	3	9	“Staffing is the largest hurdle as to why buy in is not 100%”
Not able to maintain EIP fidelity/integrity	Innovation characteristics	0	3	“Many of the tenants from the [EIP] have stopped”
Critical incidents^a^	Outer Setting^a^	1	3	“Shortage of nursing staff during pandemic”
Organizational leadership did not support the sustainment of EIP	Context	0	2	“Lack of leadership buy-in to support”
No ongoing support	Processes & interactions	1	2	“The practice has partial support and buy in”
Lack of trained personnel to continue EIP	Capacity	0	2	“Champion left position”
EIP effectiveness or benefit not observed	Innovation characteristics	0	2	“Many [staff] don’t find the [EIP] to be effective”
Unable to navigate competing demands	Processes & interactions	0	1	“Other priorities in the program”
System/policy change	Context	0	1	“Our facility is including in [new] Initiative process”
Poor collaboration/partnership	Processes & interactions	0	1	“Disconnect between [clinic] and [other] service.”
Other	Lack of adequate service users	0	1	“[Event] in use, however due to the low census, no qualifying Veterans at this time.”
No/limited funding	Capacity	0	1	“Funding [barrier]”
No ability to modify/did not modify EIP	Innovation characteristics	0	1	“Transitioning practice to a different format”
Internal/external champion did not support the sustainment of EIP	Capacity	0	2	“The [champion] is a problem who falls under our [leadership].”
Community stakeholders do not support the sustainment of the EIP	Capacity	1	1	“Buy in from some community providers has been challenging”
Total^b^		6	32	

^a^Not present in Hailemariam et al.’s factors

^b^Totals are based on 66% (39/59) of facility representatives who provided brief, open-ended text box responses in the 2021 survey. Some responders had provided more than one type of barrier

### Longitudinal pathways for practices that were fully sustained in 2021

Thirty-five percent (29/82) of facility representatives reported their practice was fully sustained in 2021, which was an average of 2.3 years (range: 1-5 years) after implementation. Of these 29 facility representatives, 76% (22/29) reported full implementation after their 6-9-months of facilitated implementation support period; the remaining facility representatives completed their implementation milestone later. Further, of these 29 who reported their practice was fully sustained, 79% (23/29) reported full sustainment at initial follow-up, which was approximately one year after the implementation period. Whereas 21% (6/29) of facility representatives who did not initially sustain their practice went on to sustain their practice by 2021.

**Fig. 3:**
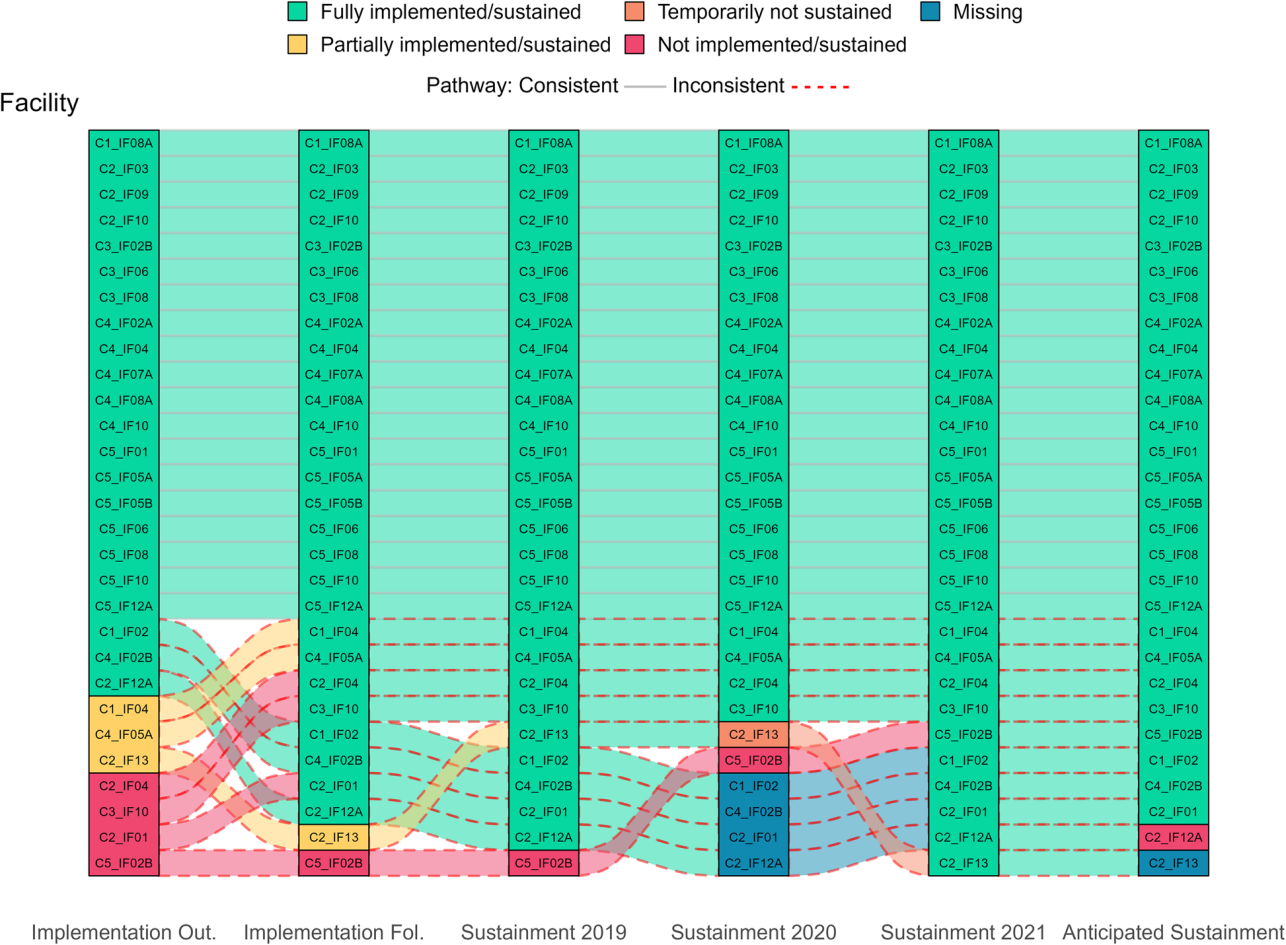
Pathways for facilities that fully sustained by 2021. *Implementation follow-up was only provided for facilities that did not fully implement their practice or were missing data. **Cohort 4 practices only have sustainment outcomes for 2020 and 2021. Cohort 5 practices only have sustainment outcomes for 2021. For the purposes of the figure visual, their statuses were carried over from earlier timepoints

Facilitators of sustainment included: demonstration of practice *effectiveness/benefit*, sufficient *organizational leadership*, appropriate *workforce*, and practice *adaptation/alignment*. Facility representatives also described potential barriers to future sustainment, including *workforce* turnover, challenges with *critical incidents* related to the COVID-19 pandemic, and concerns about *ongoing support*; they also listed potential facilitators for future sustainment that included having appropriate *workforce* and sufficient *organizational leadership* and *ongoing support*. See Tables 3 and 4 for all factors influencing sustainment. Sustained practices included more clinical interventions (45%, 13/29) and process improvements (45%, 13/29) compared to staff interventions (10%, 3/29) and were almost evenly split between the presence (48%, 14/29) or absence (52%, 15/29) of a virtual component. Figure 3 displays longitudinal pathways for facility representatives that fully sustained their practice.

#### Consistent pathways

**Fig. 4:**
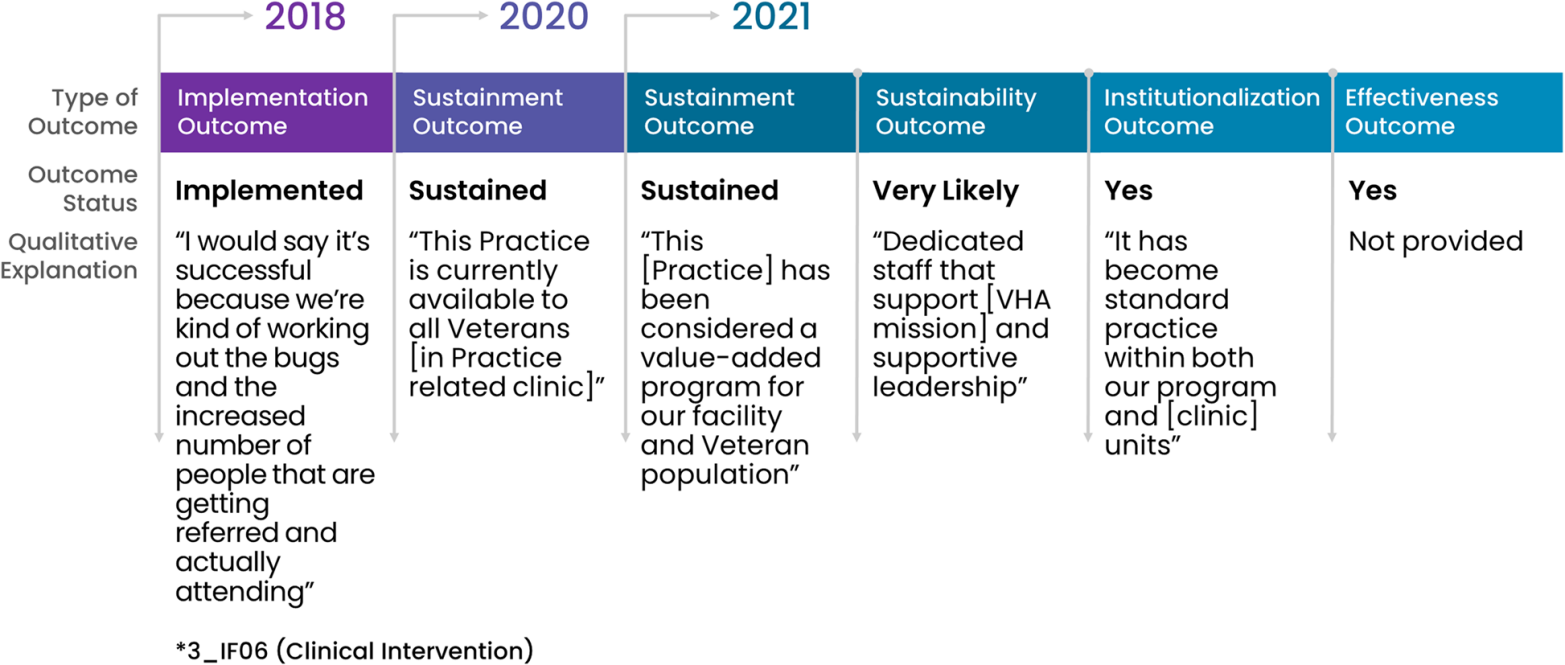
Facility with a consistent pathway to full sustainment: example outcomes and qualitative explanations

Among the 29 facility representatives who reported their practice was sustained in 2021, 66% (19/29) consistently sustained; meaning that they had sustained at all timepoints after fully implementing. All facility representatives anticipated that their practice would continue to be sustained into the future and 89% (17/19) reported their practice was institutionalized and effective. See Fig. 4 for a consistently successful pathway showing outcomes with qualitative explanations.

#### Inconsistent pathways

Among the 34% (10/29) of facility representatives with an inconsistent pathway to sustainment in 2021, the majority experienced initial challenges with implementation (60%, 6/10) compared to challenges with implementation and sustainment (10%, 1/10) or were missing data (30%, 3/10) in 2020. Facility representatives with inconsistent pathways to sustainment needed more calendar time than the 6-9-month facilitated implementation support period to overcome implementation barriers, which were often related to insufficient *workforce* and *available resources.* One facility representative also reported a temporary pause in sustainment due to *critical incidents* related to the COVID-19 pandemic that were resolved when pandemic restrictions at their facility were loosened.

**Fig. 5:**
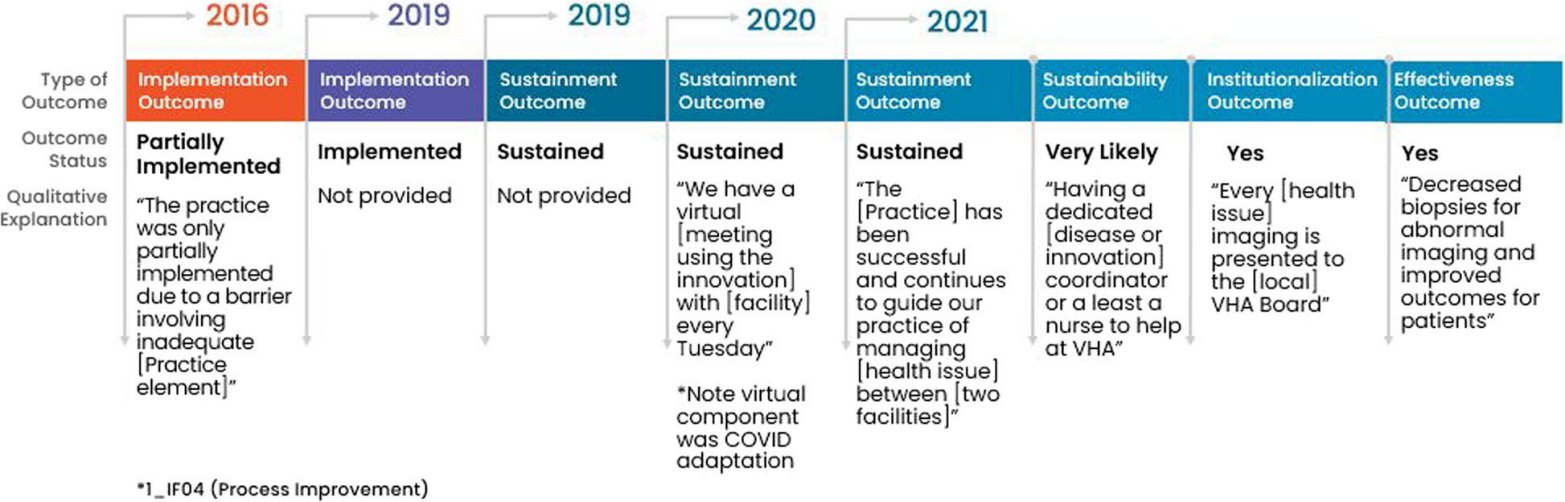
Facility with an inconsistent pathway to full sustainment: example outcomes and qualitative explanations

Once achieving sustainment, these facility representatives emphasized that facilitators to sustainment were sufficient *workforce* and *organizational leadership*. All but two facility representatives anticipated future sustainment. One facility representative anticipated future sustainment as “unlikely” without explanation and one did not respond to the question (see Fig. 3). Most (80%, 8/10) facility representatives described their practice as institutionalized and effective in 2021. Whereas, fewer (20%, 2/10) facility representatives described partial institutionalization because of *no/limited funding* or *lack of adequate number of service users* (i.e., insufficient Veteran enrollment in a voluntary program). Two out of ten (20%) facility representatives reported their practice was partially effective: one of whom cited *no/limited funding* as an issue. Figure 5 provides an example an inconsistent but successful pathway showing outcomes and qualitative explanations.

### Longitudinal pathways for practices that were not fully sustained in 2021

Thirty-seven percent (30/82) of facility representatives reported that their practice was not fully sustained because they were in a “liminal” stage [31, 32] (neither sustained nor discontinued) or permanently discontinued as of 2021, which was an average of 2.1 years (range: 1-5 years) after implementation. Only 43% (13/30) of these facility representatives reported full implementation after 6-9-months of facilitated implementation support with five additional facility representatives completing implementation later. Only 23% (7/30) of facility representatives reported full sustainment at initial follow-up, which was approximately one year after the implementation period.

**Fig. 6:**
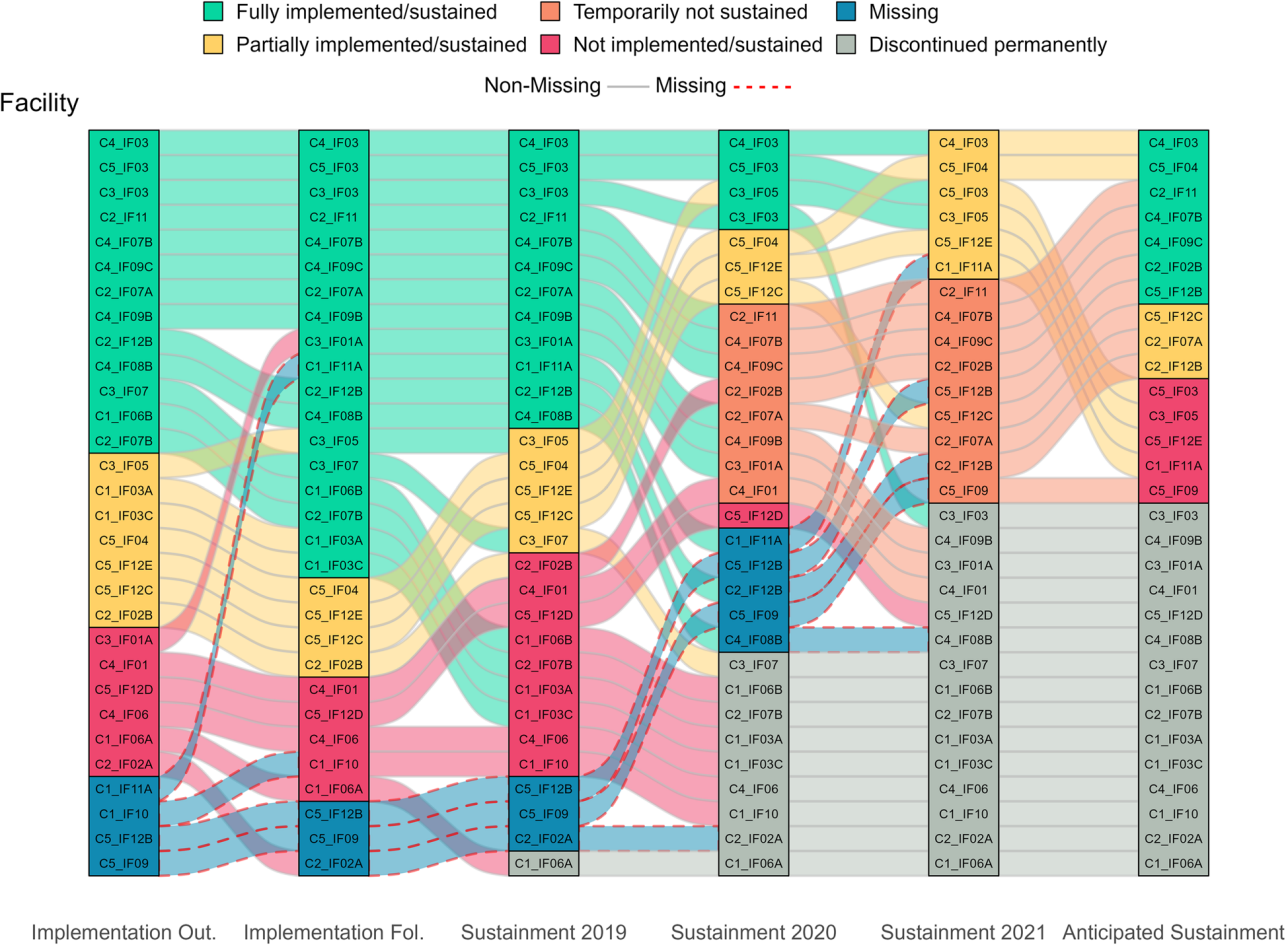
Pathways for facilities that did not fully sustain by 2021. *Implementation follow-up was only provided for facilities that did not fully implement their practice or were missing data. **Cohort 4 practices only have sustainment outcomes for 2020 and 2021. Cohort 5 practices only have sustainment outcomes for 2021. For the purposes of the figure visual, their statuses were carried over from earlier timepoints

Barriers to sustainment included: insufficient *workforce* (losing or not being able to hire staff), not being able to maintain *EIP fidelity/integrity*, *critical incidents* related to the COVID-19 pandemic, *organizational leadership did not support sustainment of EIP*, *no ongoing support*, *lack of trained personnel to continue the EIP*, and/or *EIP effectiveness/benefit was not observed*. Despite not being fully sustained, these facility representatives also described facilitators to sustainment. The most frequently reported facilitators to sustainment were *internal/external EIP champions* and *sustained/attention to topic/priority*, which were not mentioned by facility representatives with sustained practices. See Tables 3 and 4 for all factors influencing sustainment. Practices that were not fully sustained had a similar percent of clinical interventions (50%, 15/30), process improvements (43%, 13/30), and staff-oriented interventions (7%, 2/30) as those that were sustained but had fewer practices with virtual components (37%, 11/30). The following sections describe results for facility representatives who reported un-sustained practices, which is organized by status (“liminal” sustainment or discontinued permanently) and pathway (consistent or inconsistent). Figure 6 displays longitudinal pathways for facility representatives that did not fully sustain their practice.

#### Liminal sustainment

Eighteen percent (15/82) of facility representatives reported that their practices were not fully sustained because they were in a “liminal” stage of sustainment (40%, 6/15 partially sustained; 60%, 9/15 temporarily paused) since they were neither sustained nor discontinued in 2021. The major barriers associated with practices that were in a “liminal” stage of sustainment included insufficient *workforce, no ongoing support, lack of trained personnel,* and *critical incidents* related to the COVID-19 pandemic*.* Though fewer facilitators were mentioned compared to barriers, the top facilitator to sustainment was *internal/external EIP champions.*

Despite their “liminal” status, almost half (7/15) of these facility representatives were optimistic about sustaining their practice in the future. However, the remaining facility representatives did not expect to sustain their practice (33%, 5/15) or were uncertain about future sustainment (20%, 3/15). Most of these facility representatives reported their practice was not fully institutionalized (53%, 8/15 no; 27%, 4/15 partial) nor effective (20%, 3/15 no; 53%, 8/15 partial).

#### Permanently discontinued

Eighteen percent (15/82) of facility representatives reported that their practices were not fully sustained because they were permanently discontinued. Common barriers associated with practices that were discontinued included two of the same as those in a “liminal” stage (*workforce* and *critical incidents*). However, *not able to maintain EIP fidelity/integrity (top barrier), organizational leadership did not support sustainment of the EIP,* and *system policy change* were cited as other important reasons for practice discontinuation. Despite practices being discontinued, two facility representatives cited *sustained attention to topic/priority* as a facilitator.

Among facility representatives who provided responses to secondary outcomes (40%, 6/15), fifty percent (3/6) reported their practice was not fully institutionalized (33%, 2/6 no; 17%, 1/6 partial). Unexpectedly, the remaining facility representatives with discontinued practices (50%, 3/6) reported their practice was institutionalized due to some aspect of the practice becoming routinized. Regarding effectiveness, more facility representatives reported their practice was not fully effective (50%, 3/6 no; 17%, 1/6 partial). However, those who reported their discontinued practice had demonstrated effectiveness (33%, 2/6), cited they were tracking an aspect of practice effectiveness (e.g., continued using charts to show progress).

#### Consistent pathways

Only 17% (5/30) of facility representatives reported consistently less successful implementation and sustainment outcomes over time. Two out five (40%) facility representatives reported their practice being partially implemented and sustained through 2021. Despite these facility representatives’ consistent “liminal” status, responses to secondary outcomes of institutionalization, effectiveness, and anticipated sustainment were different from each other. One out of these two (50%) facility representatives reported partial institutionalization and effectiveness and anticipated full sustainment in the future, but it was dependent on having sufficient *workforce* in place. The other facility representative (1/2, 50%) reported that the practice was effective but was not institutionalized and would not be sustained in the future due to insufficient *workforce.*

**Fig. 7:**
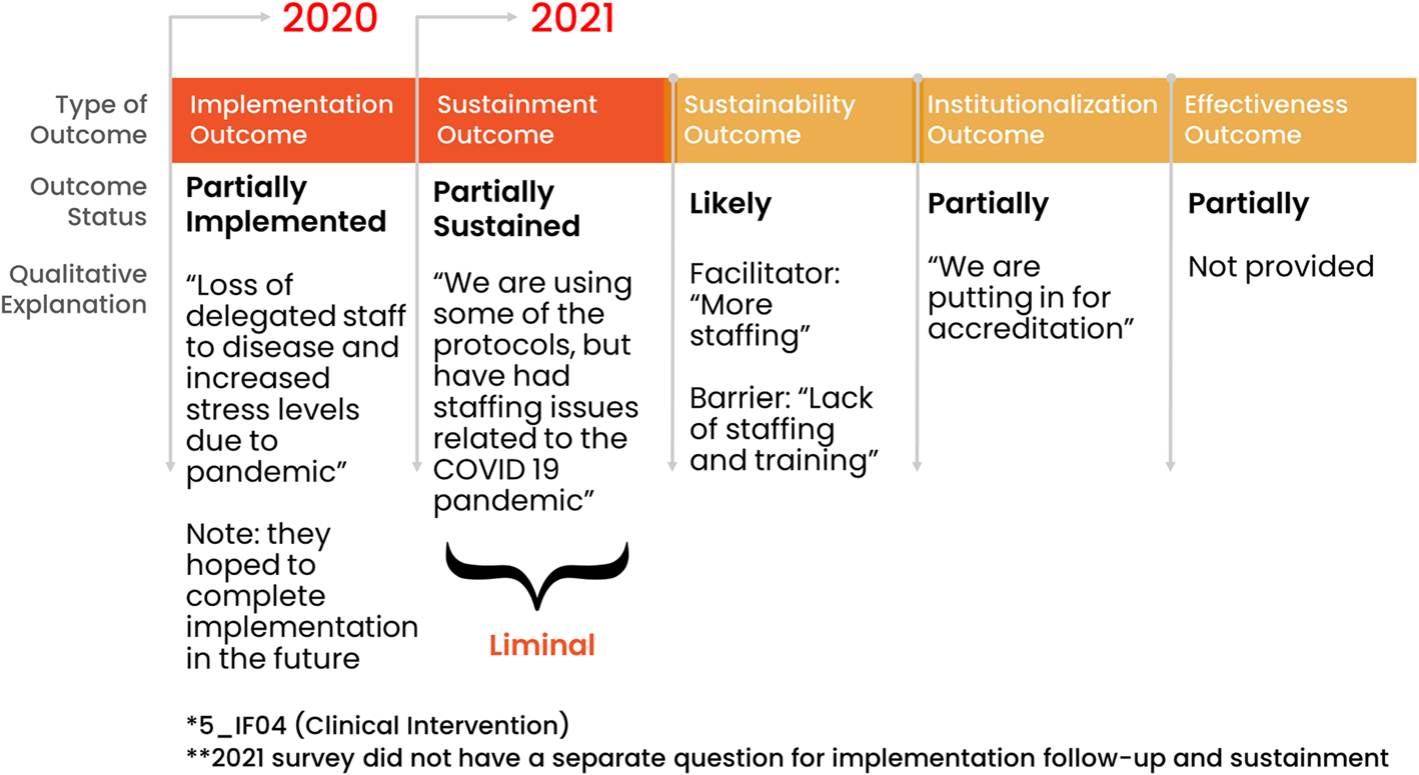
Facility with a consistent pathway to not fully sustained: example outcomes and qualitative explanations

The remaining three out of five (60%) facility representatives who were consistently less successful did not implement their practice by the end of the 6-9-months of facilitated implementation support and then reported their practice was permanently discontinued. Only 1/3 (33%) facility representatives responded to the institutionalization and effectiveness outcome questions and responded that their practice was not institutionalized nor effective. These 3/5 (60%) facility representatives experienced insurmountable barriers with implementation and never reached the sustainment phase because of *critical incidents* related to the COVID-19 pandemic or *no/limited funding*. Figure 7 provides an example pathway of a consistently unsuccessful facility showing outcomes and qualitative explanations.

#### Inconsistent pathways

Eighty-three percent (25/30) of facility representatives whose practices were not fully sustained in 2021 reported inconsistent outcomes over time, which meant their outcomes did not align over time and/or they were missing at least one outcome prior to 2021. There were two main types of inconsistent pathways leading to unsuccessful sustainment. The first type consisted of facility representatives who successfully implemented their practice, but experienced challenges with sustainment. Early on, 13/25 (52%) facility representatives reported full implementation at the end of the 6-9-months of facilitated implementation support period and another 5/25 (20%) reported full implementation with additional time when responding to the follow-up survey. Although 72% (18/25) of these facility representatives were successful at implementation, by 2021, 50% (9/18) downgraded to a “liminal” stage of sustainment (22%, 4/18 partially sustained; 28%, 5/18 temporarily not sustained) and 50% (9/18) reported being discontinued permanently.

**Fig. 8:**
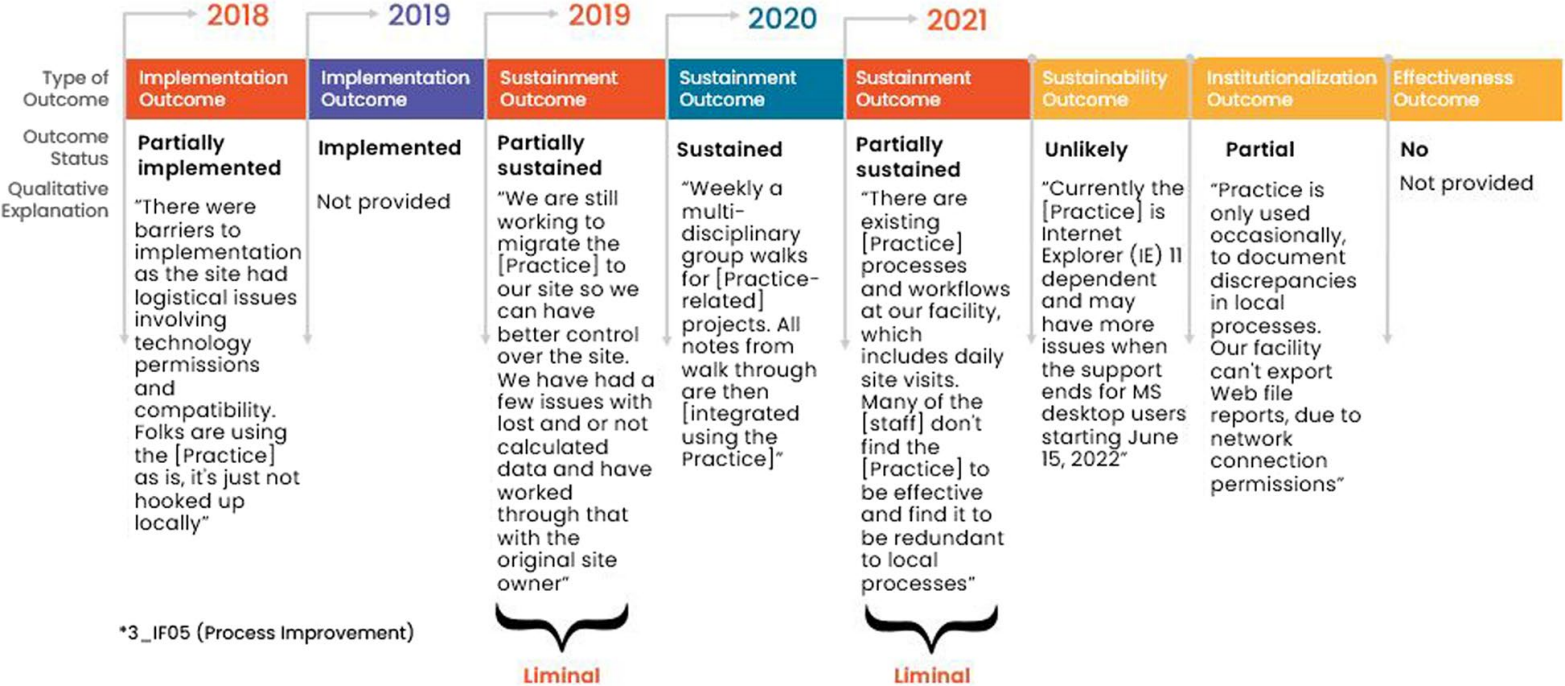
Facility with an inconsistent pathway to not fully sustained: example outcomes and qualitative explanations

The second type consisted of facility representatives (28%, 7/25) who experienced some challenges early on with implementation (42%, 3/7 not implemented; 29%, 2/7 partially implemented) or were missing implementation outcomes (29%, 2/7). These facility representatives’ status fluctuated up and down over time and by 2021, they all downgraded to being temporarily not sustained (57%, 4/7) or permanently discontinued (43%, 3/7). Figure 8 provides an example of an inconsistent not fully sustained pathway showing outcomes and qualitative explanations.

### Missing data in 2021

In 2021, 28% of facility representatives (23/82) were missing their most recent sustainment outcome. Only one out of 23 facility representatives (4%) had consistently missing implementation/sustainment outcomes over time compared to 22 (96%) facility representatives who had previously provided outcomes (i.e., inconsistent outcomes pathway due to changes over time). Of the 23 facility representatives lost-to-follow-up in 2021, only 2/23 (9%) were lost-to follow-up two years earlier in 2019. However, by 2020, an additional 12/23 facility representatives (52%) were lost to follow-up, and the remainder (9/23; 39%) had their first missing data in 2021. Facility representatives with missing 2021 sustainment outcomes had more process improvement practices (39%, 9/23) compared to staff interventions (35%, 8/23) and clinical interventions (26%, 6/23), which differed from practices that were sustained or not fully sustained. These facility representatives also had fewer practices with virtual components (30%, 7/23), which was like those that were not fully sustained.

After the 6-9-months of facilitated implementation support period, only 17% (4/23) of facility representatives had missing implementation outcomes data. However, three out of four (75%) facility representatives responded at follow-up. One of these three facility representatives reported their practice was implemented and sustained before being missing in 2021. Whereas the other two other facility representatives reported at follow-up that their practice was not implemented nor sustained before being missing in 2021.

**Fig. 9:**
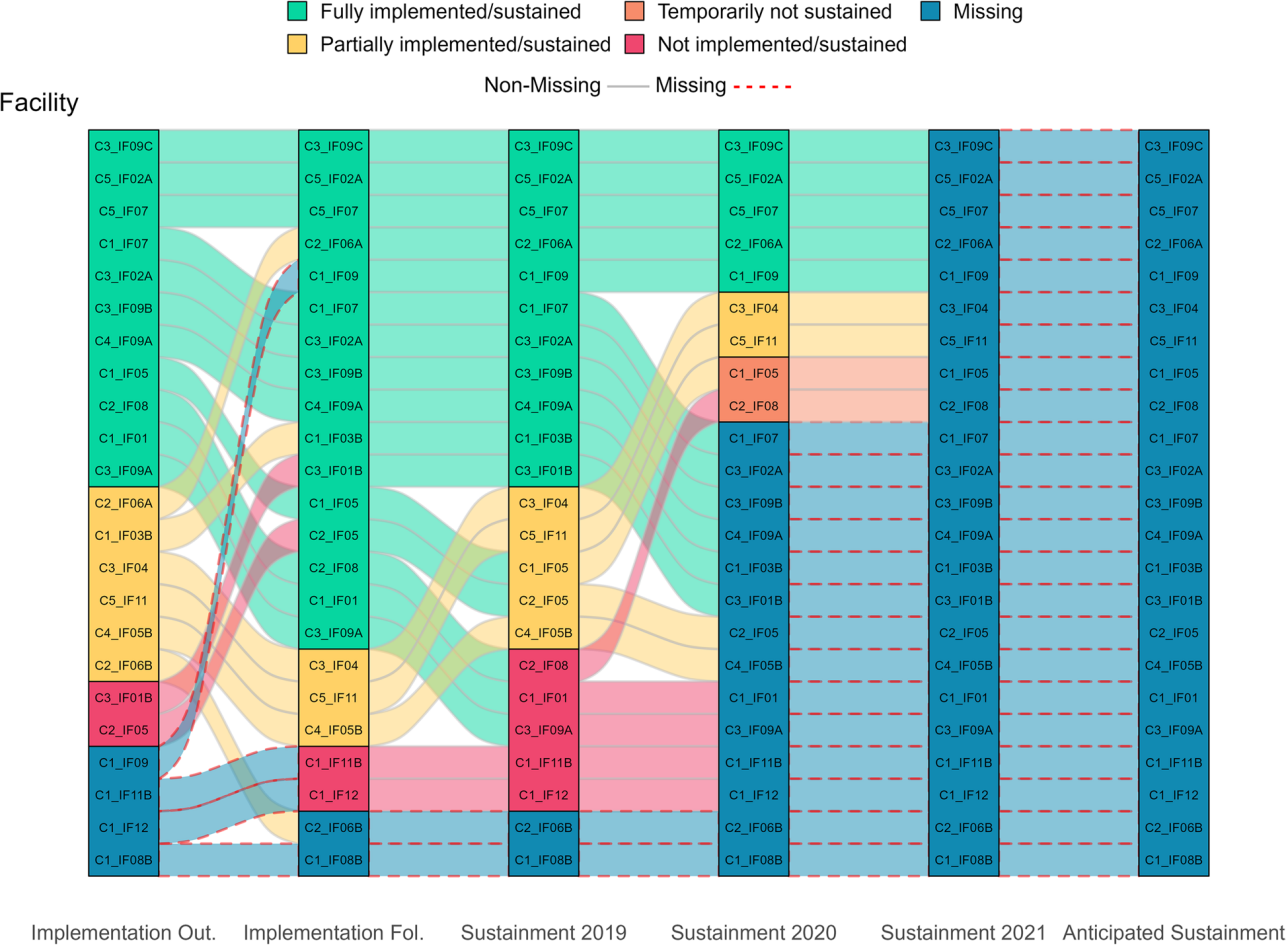
Pathways for facilities with missing data in 2021. *Implementation follow-up was only provided for facilities that did not fully implement their practice or were missing data. **Cohort 4 practices only have sustainment outcomes for 2020 and 2021. Cohort 5 practices only have sustainment outcomes for 2021. For the purposes of the figure visual, their statuses were carried over from earlier timepoints

Seventy-percent (16/23) of facility representatives with data missing in 2021 reported they fully implemented by the time of the second implementation assessment. Although these facility representatives were lost-to-follow-up in 2021, most (68%, 11/16) reported full implementation or sustainment as their last known status. The remaining five facility representatives reported a downgraded status of being temporarily (13%, 2/16), partially (6%, 1/16), or not (13%, 2/16) sustained before being lost-to-follow-up in subsequent assessments. See Fig. 9 for pathways of facilities with missing outcomes in 2021.

#### Missing data overall

With respect to missing data trends across all 82 facility representatives, 41% (34/82) percent had at least one missing time point across all years of data collection with an average of 1.8 missing time points overall. Only 1/82 (1%) facility representative was consistently missing outcomes for all time-points. Among the 5/82 (6%) facility representatives with two missing timepoints in a row, only 40% (2/5) responded to subsequent surveys. Facility representatives from Cohort 1 (53%, 9/17) and Cohort 3 (43%, 6/14) had more missing data than those from other Cohorts (Cohort 2 = 24%, 4/17; Cohort 4 = 19%, 3/16; Cohort 5 = 17%, 3/18).

## Discussion

### Longitudinal pathways are often non-linear

Our evaluation includes implementation and sustainment outcomes from 82 different VHA facilities over 4 timepoints, spanning 1-5 years after 6-9-months of facilitated implementation support (including 1 facility with missing data across all assessment points). It has long been thought that initial implementation success predicts future success [33–35]. However, in this evaluation, we found most (70%) facilities had non-linear (inconsistent or changing outcomes) longitudinal pathways as their practices transitioned from implementation to long-term sustainment compared to 30% of facilities that had linear (consistent or same outcomes) longitudinal pathways from implementation and sustainment [14].

Among the 29 facilities with practices that were sustained in 2021, two-thirds had consistently reported sustainment for prior timepoints. This finding is affirmed by published literature emphasizing that initial success leads to future success [8, 21]. Facility representatives with practices that were consistently successful over time often led to EIPs that were highly institutionalized. However, in contrast to published literature, we also found that one-third of facilities had delayed implementation, but eventually fully implemented and sustained their practice.

Given these findings, we suggest that evaluators and operational partners consider incorporating an equity lens when choosing which facilities and practices to support. Specifically, do not give up on those that do not meet initial milestones because facilities with initial struggles can persevere and sustain over the long-term. Additionally, it is important to recognize that facilities that meet initial milestones can also experience challenges and fail to sustain their practice over the long-term.

### Sustainment benchmarks are not well established

Published literature highlights that measuring sustainment is challenging, contributing to few studies reporting long-term sustainment outcomes over time [5]. There are no well-established sustainment benchmarks, so it is difficult for researchers/evaluators to determine how their sustainment rates compare with others [8]. Wiltsey-Stirman et al. describe in their systematic review that partially sustained EIPs were reported more often than sustained, even if initial implementation was successful [8]. Conversely, facility representatives in this evaluation reported relatively high sustainment rates (35%) compared to those in a “liminal” sustainment stage (18% were partially sustained/temporarily paused). Wiltsey-Stirman et al. also state “virtually no studies reveal the nature of the changes made, the reasons for the changes, or the process by which adaptations or decisions to discontinue elements of the program or intervention were made.” [8] Building on Wiltsey-Stirman et al.’s systematic review, Hailemariam et al.’s systematic review identified 26 facilitating and 23 hindering factors impacting sustainment, which were then mapped onto 4 major thematic areas of the influences on sustainment framework (innovation characteristics, context, capacity, processes and interactions) [5, 8].

To enhance knowledge about influences on sustainment outcomes, we mapped facility representatives’ open-ended descriptions of barriers and facilitators onto Hailemariam et al.’s factors (Tables 3 and 4) [5, 8]. We extend Hailemariam et al.’s work by linking barriers and facilitators to actual sustainment outcomes. The top 3 facilitators to sustainment that facility representatives mentioned included *EIP effectiveness/benefit* (innovation characteristics); *organizational leadership* (context), and *workforce* (capacity), which differed somewhat from Hailemariam et al.’s top 3 identified in their systematic review (*adaptation* (processes and interaction), *funding* (capacity), *organizational leadership* (context)).

The top three barriers to sustainment identified by facility representatives in this evaluation were *workforce* (capacity), *not able to maintain EIP fidelity/integrity* (innovation characteristics), and *critical incidents* due to the COVID-19 pandemic (outer setting), which we added based on the updated CFIR [30]. Conversely, Hailemariam et al.’s most frequent barriers to sustainment were *no/limited funding* (capacity), *lack of resources* (capacity), and being *unable to navigate competing demands* (processes and interaction). We identified an additional facilitator referred to as *sustained attention to topic/priority*, which occurred when elements of a practice were integrated into workflow despite practice discontinuation. Of note, *sustained attention to topic/priority* is not included in Hailemariam et al.’s systematic review, but is referenced in other sustainment literature [27]. Given our evaluation was conducted within a single healthcare system and during the COVID-19 pandemic, it is not surprising that our top barriers and facilitators differ from Hailemariam et. al.’s systematic review. Since facility representatives reported challenges 2 years (on average) after implementation, understanding the timing and factors that contribute to successful and unsuccessful sustainment can provide DoE with specific strategies and opportunities to intervene to improve its program.

### Lessons learned from evaluating longitudinal sustainment

In the following sections, we describe lessons learned that will enhance evaluation of a complex array of practices as they transition from initial implementation to longer-term sustainment at each facility. We also provide recommendations to improve sustainment reporting and measurement using pragmatic measures to help enhance the field of implementation science.

#### Maximizing open-ended text boxes

We purposefully administered a pragmatic sustainment survey (i.e., easy to understand, quick to complete, non-specific to practices) since it was more feasible in a real-world setting when comparing diverse EIPs [3, 36]. As part of our survey, we included open-ended text boxes to offer facility representatives opportunities to explain and contextualize their sustainment outcomes. In our evaluation, new insight on factors influencing sustainment depended on responses to open-ended text boxes in the survey. Closed ended-survey items can be limiting because they rely on a priori assumptions and in our case, did not generate the level of contextual information needed to discern all the reasons or types of sustainment/discontinuation. For example, through participant responses to open-ended text boxes, we learned that a practice that is temporarily sustained because of a short-term barrier, (e.g., the COVID-19 pandemic lockdowns, temporary hiring freeze, etc.), is different than a facility with a practice that is partially sustained, (e.g., only some of the practice components are in use or it is only being used some of the time). As a result, open-ended text boxes in our survey allowed us to reclassify response options, which also led to creating a new response option (temporary hold outcome) in our survey to better reflect facility representatives’ experiences and improve validity of responses. Overall, we found that a high proportion of facility representatives responded to open-ended text boxes and qualitative data collected in the survey was invaluable to contextualizing outcomes, understanding salience of determinants, and describing changes in outcomes at a facility [37, 38]. Learning about sustainment experiences in a facility representative’s own words allows for insight into their thought process interpreting questions, as well as a rationale behind their response.

In future surveys, we aim to leverage responses to open-ended text boxes (e.g., making some mandatory, highlighting their importance). This is especially important for practices that were discontinued but participants revealed that elements were integrated into other aspects of workflow and processes. These facility representatives reported that while the practice was not sustained, there was institutionalization and/or effectiveness. As described in the literature as “sustained change”, completing implementation had an impact on the health topic/priority even though it was no longer sustained, demonstrating some lasting effects/benefits even when practice is no longer in use [27]. This result also illustrates that relying on sustainment rates alone may lead to missed opportunities in understanding important nuances of sustainment contexts and the full continuum of sustainment (i.e., in some cases (negative outcomes) it may be important to measure impacts in other ways).

#### Minimizing missing data

Missing data is common challenge in longitudinal studies [39]. In our longitudinal evaluation, missing data hindered our ability to fully understand outcomes and describe each longitudinal pathway. One might assume that participants are less likely to respond if they are not sustaining. However, we found that most (69%) facility representatives reported that they had implemented and sustained their practice at their last known status. Thus, a missing timepoint may not mean that the practice is discontinued. Therefore, it is important for evaluators to follow-up on those with a missing timepoint. In our evaluation, we found that less than half of facility representatives (40%) responded after two consecutive missing time points. As a result, we will consider facility representatives lost-to-follow-up (removed from the sample) after three consecutive years of missing data. Sometimes, a change in the primary point-of-contact can result in missing data. To reduce missing data and to track changes in staffing, we include a line in the recruitment email about providing contact information for current facility representatives.

When following-up with participants longitudinally over a longer time-period, engagement may decrease because of insufficient rapport [40]. Obtaining participant input is challenging throughout longitudinal studies. However, it is important to develop ways to engage participants over the long-term to help minimize missing data. Teague’s systematic review and meta-analysis suggest reducing participant burden overall rather than incorporating a larger number of strategies to improve retention [41]. As we move forward with our evaluation, we aim to incorporate new strategies that will not burden facility representatives, such as using instant messaging instead of email follow-ups since it helps to improve recruitment rates [25], extending recruitment periods or changing the recruitment season, offering incentives if employees complete surveys outside of work hours, and eliciting feedback from participants about how to increase response rates. Our literature search for “sustainment” and “missing data” resulted in zero articles. Therefore, strategies for reducing missing data and analyzing missing data is an important area for future development in the sustainment literature.

#### Strengthening survey questions

Moving forward, we aim to improve our survey in important ways. We will do more robust testing using cognitive interviews to ensure questions and responses resonate with participants [42, 43]. For example, our question about measuring effectiveness was originally “what indication do you use to determine its effectiveness”, but upon reviewing participant responses, we realized that while we want to know the measure they use, we also want to understand their perception of effectiveness and how it was determined (i.e., actual data or self-reported based on experience/feedback).

Additionally, we included Likert Scale determinant questions with open-ended text boxes that were informed by the literature [26–28, 44]. However, we will discontinue these questions because the response quality was poor, and we were unable to determine the salience of determinants. Lower quality responses affirm literature reporting on increased question complexity (content and intensity) and cognitive burden for respondents for Likert Scale questions [45]. We had better quality responses to multiple choice questions (i.e., outcome questions) and the barrier and facilitator open-ended text boxes. Building off literature on alternatives to Likert Scale questions [45] and the pragmatic PReSS sustainment scale mentioned earlier [11], we will use simplified language and replace Likert Scale determinant questions with an open-ended barrier and facilitator text box. In addition, we will add phrase completion [45] questions that focus on not only the “what” practice components are sustained, but also on the “who” has sustained, “when” the practice is sustained, and “where” the practice is sustained. These questions will provide a pragmatic yet nuanced understanding of sustainment, especially since we learned in this evaluation that sustainment is not binary and that practices can be in a “liminal” stage of sustainment or on the cusp of sustaining or discontinuing [27]. Given the changing nature of sustainment, we will continue including the question about sustainability (anticipated outcome) for all participants unless they indicate that their practice is permanently discontinued.

Qualitative responses were crucial in understanding a facility representative’s practice status, as well as the salience of barriers and facilitators. To improve qualitative response rates and quality, we will include language explaining the importance of specific open-ended questions, increase the size of text boxes, and note that responses are not limited by the size of the text box [46]. Further, we will reduce the overall survey length and require (i.e., make the question not skippable) a set number of questions deemed most relevant for this evaluation, which include a primary sustainment outcome, sustainability outcome, and “please explain” qualitative questions providing insight on contextual factors that help or hinder sustainment.

## Limitations

This evaluation has limitations. First, we compared sustainment outcomes longitudinally for 82 facilities and 57 practices, which is simultaneously a strength and limitation. It is a strength because we report on diverse practices across a large, integrated health care system, thus, prompting us to use a pragmatic approach that can be applied across different types of practices. However, comparing sustainment outcomes of diverse practices limited our ability to use more specific sustainment measures (i.e., fidelity checklists) or assess intra-group variation within a single practice. Second, missing data is a common challenge in longitudinal studies in general and is an ongoing challenge in understanding longitudinal patterns of facilities’ sustainment of DoE’s practices. Only one facility was missing outcomes for all time points. Even though about 1/3 of facilities were missing recent outcomes, we still had valuable information about their prior implementation and sustainment outcomes (e.g., most facilities were previously successful). Moving forward, we aim to incorporate new techniques described above for minimizing missing data. Third, the 82 facilities adopting a DoE practice represent several cohorts, each beginning in a different year resulting in varying numbers of data collection timepoints. Facility representatives with practices in the earliest cohorts had lower response rates in 2021, which might be due to survey fatigue, staff/point-of-contact turnover, changes in facility priority, and/or reduced engagement with DoE. Fourth, the barriers and facilitators in Tables 3 and 4 were based on optional open-ended text responses in the 2021 survey. Given the challenges, changes, and complexities of longitudinal datasets (e.g., missing data) it was not feasible to compare barriers and facilitators from previous years. Moving forward, we will require responses to a select number of open-ended text boxes. Fifth, we relied on self-report via interviews and surveys to assess outcomes, which may be less objective than in-person observation. However, self-report it is commonly used when studying sustainment because it is more pragmatic and feasible [3]. Given this is a real-world, pragmatic evaluation that spanned from 2016-2021, we worked with our operational partners to determine the best ways to obtain sustainment outcomes within the constraints of our project funding and without overburdening busy, frontline VHA employees.

To further expand on our evaluation, areas for future work could include: 1) understanding how Shark Tank criteria may impact practice implementation and sustainment outcomes; 2) using qualitative methods to collect more in-depth knowledge of barriers and facilitators to long-term sustainment; 3) determining if facilities without DoE support adopt practices and if there are differences in outcomes between those with and without DoE support; and 4) assessing if facilities with practices in a “liminal” stage benefit from additional support or intervention.

## Conclusions

We enhance existing literature with our longitudinal analyses of multiple implementation and sustainment-related outcomes plus lessons learned from the large-scale DoE program in VHA to disseminate EIPs. The relatively high rates of practices with long-term sustainment provide important evidence to support that VHA’s DoE is achieving its goal as a sustainable model for large learning health systems. Additionally, DoE has demonstrated longevity within VHA, founded in 2016 and persisting through three administrations and shifting priorities. We also highlight the importance of understanding the dynamic, longitudinal pathways that practices often undergo from implementation to longer-term sustainment. We offer novel suggestions and lessons learned from our evaluation to inform the field of implementation science and to strengthen future efforts in understanding longitudinal sustainment of EIPs.

### Supplementary Information

**Additional file 1.** Diffusion of Excellence Evaluation and Practice Description.

**Additional file 2.** Full Sustainment Survey.

**Additional file 3.** Sustainment outcome by practice type. Sustainment outcome by presence/absence of a virtual component. Sustainment outcome by sustainability outcome.

## Data Availability

The datasets generated and/or analyzed during the current evaluation are not available due to participant privacy but may be available from the corresponding author on reasonable request.

## Abbreviations

CFIR: Consolidated Framework for Implementation Research
DoE: Diffusion of Excellence
EIP: Evidence-informed practice
VHA: Veterans Health Administration
