# Supplementary material for: Understanding pathways from implementation to sustainment: a longitudinal, mixed methods analysis of promising practices implemented in the Veterans Health Administration
Source: Implement Sci. 2024 May 7;19:34. doi: 10.1186/s13012-024-01361-z (PMC11075255; doi:10.1186/s13012-024-01361-z)
Supplement: Supplementary file 1 — Additional file 1. Diffusion of Excellence Evaluation and Practice Description. [file 13012_2024_1361_MOESM1_ESM.docx]

# Additional File 1: Veterans Health Administration (VHA) Diffusion of Excellence (DoE) Program and the Spreading Healthcare Access, Activities, Research and Knowledge (SHAARK) Partnered Evaluation Initiative (PEI)


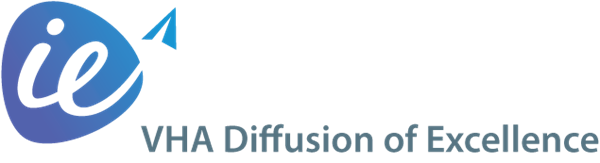


## VHA Diffusion of Excellence

The [Veterans Health Administration](https://www.va.gov/health/) (VHA), the largest integrated health system in the United States (US), provides care at over 1,000 health care facilities, including medical centers and outpatient care sites, serving approximately 9 million enrolled US Military Veterans each year. In October of 2015, the VHA developed the [Diffusion of Excellence](https://www.innovation.va.gov/ecosystem/views/diffusion-excellence/doe.html) (DoE) program to identify, replicate, and scale evidence-informed practices (EIPs) across the VHA. The DoE is housed within the VHA [Innovation Ecosystem](https://www.innovation.va.gov/ecosystem/views/home.html), part of the VHA [Office of Healthcare Innovation and Learning](https://www.innovation.va.gov/hil/home.html) (OHIL) within the [Office of Discovery, Education and Affiliate Networks](https://www.va.gov/dean/) (DEAN). DoE is currently directed by Blake Henderson. More information can be found on the websites linked above or by emailing [VADiffusionSupport@va.gov](mailto:VADiffusionSupport@va.gov). The DoE includes four distinct phases:

**VHA employees develop evidence-informed practices (EIPs)**

VHA employees develop and implement innovative practices in their local facility; if the innovation is successfully implemented with measurable positive impact for the system, employees, or patients, then the lead developer (a VHA employee or team) can submit their practice for consideration in the Shark Tank in Phase 2. In order to be considered, innovations must align with a VHA high priority area, e.g., Access, Care Coordination, or Veteran Experience.

**EIPs are selected and compete in the VHA Shark Tank Competition**

A governance board, comprising national executive-level VHA leaders and subject matter experts, reviews the practices submitted and approves a shorter list of finalists who are entered into the [Shark Tank Competition](https://www.innovation.va.gov/ecosystem/views/diffusion-excellence/sharktank.html). Finalists develop a 5-minute pitch describing their innovation and the resources required to implement and deliver the innovation.

VHA facility or network directors, who volunteer to participate as a “Shark” in the Shark Tank, listen to pitches and compete by placing bids on one or more practices that they want to implement in their facility/network. These bids typically include key resources (e.g., staff, office space, travel funds), and if a Shark’s bid is selected, the facility wins approximately 6 months of support from an Implementation Facilitator.

The DoE governance board reviews bids and selects a final list of practices that are designated Promising Practices that are matched with at least one facility or network for implementation.

**EIPs that win in Shark Tank are replicated in additional facilities**

Each facility or network identifies an “Implementing Fellow,” a VHA employee who leads implementation of the practice. The Implementing Fellow attends a “Diffusion Base Camp” to meet with Diffusion Fellows (the VHA employee who developed the practice) and an Implementation Facilitator, who provides project management expertise, including coordinating weekly meetings and tracking tasks and milestones. During Base Camp, teams attend plenary sessions about implementation strategies and develop a plan to implement their practice. After the replication period ends, teams reconvene for a “Diffusion Summit” to discuss implementation experiences and lessons learned to inform implementation at additional sites.

**EIPs are selected for a diffusion pathway**

The DoE governance board reviews practice replications and determines the most appropriate diffusion pathway for each practice:

[National Diffusion Practices](https://www.innovation.va.gov/ecosystem/views/diffusion-excellence/national-diffusion-practices.html#:~:text=Diffusion%20of%20Excellence%20nationally%20implements%20select%20Promising%20Practices,provides%20resourcing%20for%20a%20three-year%20national%20implementation%20effort.): Practices that demonstrate *very high* impact, replicability, stakeholder support, and/or cost effectiveness are assigned a Diffusion Specialist and further resourcing for an approximately 3-year national implementation effort. As of May 2023, DoE has provided support of for 12 National Diffusion Practices.

Initial Diffusion Practices: Practices that demonstrate *strong* impact, replicability, stakeholder support, and/or cost effectiveness are invited to the VHA Diffusion Academy to develop a diffusion strategy for the next 1 – 2 years.

Organic Diffusion Practices: Practices that demonstrate *moderate* impact, replicability, stakeholder support, and/or cost effectiveness are made available on the [Diffusion Marketplace](https://marketplace.va.gov/) (along with National and Initial Diffusion Practices and innovations originating from other VHA programs) for organic dissemination and are eligible for ad hoc support.

The table below describes the DoE Promising Practices that were the focus of sustainment measurement in the present paper.

Cohort 1 – 5 Promising Practice Descriptions

| **Cohort 1 Promising Practice Descriptions^1^** | | | | |
| --- | --- | --- | --- | --- |
| **Number** | **Practice Type^2^** | **DoE Category** | **Name** | **Short Description** |
| 1 | Process Improvement^3^ | Access | Access Data Dashboard to Improve Clinic Management | This practice compiles all access metrics (formatted similarly) in a single sheet to highlight metrics meeting/not meeting targets for end-users and leaders. The dashboard is posted on SharePoint and displayed in a public venue (a highly utilized conference room). |
| 2 | Process Improvement^3^ | Access | Improving Same Day Access Using RNCM Chair Visits | This practice integrates RN Care Managers (RNCMs) into PACT Teams and better leverages their Scope of Practice (SOP). Clerical Associates schedule same day patient appointments with RNCMs when there is no PCP availability (and not otherwise according to the process map). RNCMs triage patients and refer to the Emergency Department (ED) or Primary Care (PC) if treatment is not within their Scope of Practice (SOP). |
| 3 | Process Improvement^3^ | Access | Increasing Access to Primary Care: Utilize Multi-modal Approaches and Advance Pharmacy Practice in Primary Care Setting | This practice integrates Clinical Pharmacist Specialists (CPSs) into PACT Teams and better leverages their Scope of Practice (SOP). The CPS converts PCP appointments into CPS appointments when appropriate (CPS Point-Of-Contact Program). In addition, the CPS completes a phone call visit with new patients prior to their PCP visit, and an accurate medication list is obtained, reconciled, and interventions are made to optimize medication use and safety (New Patient Medication Intake Clinic). This practice also includes an educational campaign about pharmacy resources available to PCPs for chronic disease state management (Medication Management Clinic Educational Campaign). |
| 4 | Process Improvement | Access | The Journey to Open Access in Primary Care at VACCHCS: Achievement and Sustainment | This practices highlights key processes for Open Access in Primary Care. |
| 5 | Process Improvement^3^ | Care Coordination | eScreening Program | This practice utilizes a mobile tool (iPad) that allows Veterans to report their mental and physical health symptoms directly to the Computerized Patient Record System (CPRS) from the waiting room, instead of completing a pen and paper screen during the PCP visit. |
| 6 | Process Improvement^3^ | Care Coordination | Regional Liver Cancer Tumor Board | This practice allows Gastroenterology and Oncology providers at CBOCs to submit Hepatocellular Carcinoma patient cases via SharePoint to a Liver Cancer Tumor Board consisting of hepatology, oncology, surgery, radiology, interventional radiology, palliative care, and radiation therapy providers for consultation. |
| 7 | Staff Intervention | Employee Engagement | Unit Tracking Board | This practice compiles core measures (refined to the simplest form) on a reusable tracking board that can be used in any department/unit. The tracking board is posted in a public venue and updated regularly to provide data to individuals involved in direct Veteran care, e.g., nurses. |
| 8 | Staff Intervention | Employee Engagement | Using External (Non-VA) Comparative Data to Achieve Excellence and Engage Employees | This practice shares both VA and non-VA non-clinical data with employees to help them set goals for improvement. |
| 9 | Process Improvement | Quality and Safety | Code Tray Redesign | This practice utilizes a reorganized code tray with medication in alphabetical order and placed face up in a custom fitted foam tray. All medication comes from the same lot number and expiration date. |
| 10 | Process Improvement^3^ | Quality and Safety | Flu Self-Reporting Desktop Icon to Capture Employee Vaccinations Received Outside the VA | This practice utilizes an Info Path form that can be accessed by clicking the Flu Icon on every laptop or desktop workstation. The Info Path form is linked to SharePoint and Occupational Health gets the report and documents it into the Occupational Health Reporting System. |
| 11 | Clinical Intervention | Quality and Safety | Planning for Future Medical Decisions via Group Visits | This practice utilizes Advanced Healthcare Planning (AHCP) group visits to provide Veterans with the opportunity to interact with a specialty trained health professional regarding AHCP and Advance Directives (ADs). |
| 12 | Process Improvement | Quality and Safety | WAKE Score for Recovery from Anesthesia Sedation | This practice utilizes redesigned anesthesia to meet the WAKE Score. |
| **Cohort 2 Promising Practice Descriptions** | | | | |
| **Number** | **Practice Type** | **DoE Category** | **Name** | **Short Description** |
| 1 | Clinical Intervention | Access | Home-Based Mental Health Evaluation (HOME) Program | This practice provides clinical services during the transition from inpatient to outpatient mental health care. |
| 2 | Clinical Intervention^3^ | Access | National TeleWound Care Practice | This practice provides expedited remote diagnosis and treatment of various types of wounds via clinical video telehealth, e-Consults, and Store-and-Forward telehealth. |
| 3 | Process Improvement | Access | Improving Access through Consult Triage | This practice requires physicians to review consults at the time of the order request and triage patients appropriately. |
| 4 | Clinical Intervention | Care Coordination | VHA Rapid Naloxone | This practice expands the availability of Narcan Nasal Spray by distributing it to VA Police and Veterans, and putting it within some Automated External Defibrillator (AED) cabinets. |
| 5 | Staff Intervention^3^ | Care Coordination | Women's Health Mini-Residency | This practice uses a pre-set curriculum from the Office of Women's Health Education to increase the volume of clinicians competent to care for female Veterans. |
| 6 | Staff Intervention^3^ | Employee Engagement | EMS Competency Tracker Program | This practice allows users to track employee performance, quickly identify competent coverage for callins/no-shows and employees on leave, and assess new areas to focus training using a custom board. |
| 7 | Staff Intervention | Employee Engagement | Jump Start Program for New Employees | This practice establishes a dedicated new hire welcome website and the expansion of the facility-coaching program to include matching new hires with experienced coaches. |
| 8 | Staff Intervention | Employee Engagement | Veterans Engagement Day | This practice offers staff, who do not normally interact with Veterans, the opportunity to spend a day meeting Veterans and hearing their stories. |
| 9 | Process Improvement^3^ | Quality and Safety | E-Consult Implementation: Novel Screening and Transfer Process for Patients Seeking Substance Detoxification | This practice uses an enhanced screening and transfer algorithm (based on available literature and multidisciplinary consensus) that is embedded in a novel E-consultation order set for providers to use with patients seeking substance detoxification. |
| 10 | Clinical Intervention | Quality and Safety | Project HAPPEN: Hospital Acquired Pneumonia Prevention by Engaging Nurses | This practice engages nursing staff to provide oral care to Veterans. |
| 11 | Clinical Intervention | Veterans Experience | Chaplain Groups for Veterans with Moral Injury | This practice is a group visit for Veterans diagnosed with Posttraumatic Stress Disorder (PTSD) and struggling with service-connected moral injury. |
| 12 | Clinical Intervention | Veterans Experience | My Life, My Story | This practice involves interviewing Veterans to capture their life story and adding it to their VA Electronic Health Record for VA providers to read. |
| 13 | Clinical Intervention | Veterans Experience | Pain University | This practice is a comprehensive university-like program that offers ~30 classes and treatment labs to Veterans related to pain. |
| **Cohort 3 Promising Practice Descriptions** | | | | |
| **Number** | **Practice Type** | **DoE Category** | **Name** | **Short Description** |
| 1 | Process Improvement | Access | A Best Practice: The Collaborative Consult Scheduling Model | This practice initiates a partnership between services to create a dedicated team of Advanced Medical Support Assistants (AMSAs) to focus on consult scheduling; the team can make one call and address several consults for a single Veteran. |
| 2 | Process Improvement^3^ | Access | VistA Automation for Prosthetics | This practice automates the home oxygen billing process by integrating Microsoft Excel and Veterans Information Systems and Technology Architecture (VistA); staff no longer need to print the bill and complete lengthy reviews, allowing for employees to improve access elsewhere in Prosthetics. |
| 3 | Clinical Intervention | Care Coordination | HBPC Interdisciplinary Project to Reduce Hospitalization of Veterans with COPD, CHF, and Pneumonia | This practice uses a multidisciplinary team to decrease hospitalizations by educating caregivers and patients on disease management using Zone Charts and coordinating care with community providers. |
| 4 | Clinical Intervention | Care Coordination | VIONE | This practice is a model for clinical pharmacists to review each patient's medication profile to identify appropriate medications to de-prescribe. |
| 5 | Process Improvement^3^ | Quality and Safety | SharePoint Construction Safety Tool for All VHA Facilities | This practice provides an electronic means to document construction safety inspections using SharePoint; the tool provides a mechanism to track deficiencies from identification to corrective action, ensuring compliance with federal regulations. |
| 6 | Clinical Intervention | Quality and Safety | Substance Use and Suicide Prevention Group Therapy Module | This practice is a one-hour psychoeducational group therapy session where a SUD treatment specialist and suicide prevention coordinator cover the prevalence of suicide in SUD treatment populations. |
| 7 | Clinical Intervention | Veterans Experience | Community Housing Fair | This practice is a “one-stop shop” event to help homeless Veterans achieve permanent housing status in less than eight hours. |
| 8 | Process Improvement | Veterans Experience | FLOW3 | This practice is a workflow management system that incorporates three custom-designed features to address issues with the process for authorization of artificial limbs: an artificial limb consult template, a consult comment tool, and a web-based dashboard with custom screens for staff member workflow management. |
| 9 | Clinical Intervention | Veterans Experience | Partnering with Veteran Service Agencies and Faith-Based Organizations to Prevent Veteran and Service Member Suicides | This practice trains clergy and their congregations to identify and refer at-risk Veterans and Service Members to VA care. |
| 10 | Process Improvement | Veterans Experience | VeTRANS - Veteran Transportation Project | This practice is a collaborative solution between Voluntary Service, Social Work Service, and Health Administration Service that provides transportation to Veterans that are discharged from acute care or the emergency room and do not qualify for Beneficiary Travel or have a viable form of transportation. |
| **Cohort 4 Promising Practice Descriptions** | | | | |
| **Number** | **Practice Type** | **DoE Category** | **Name** | **Short Description** |
| 1 | Clinical Intervention | Access | COACH - Caring for Older Adults and Caregivers at Home | This practice is a home-based dementia care program that serves Veterans with dementia and their caregivers. The program provides dementia care to Veterans in their home and offers education in dementia care and resource assistance to the Veteran caregivers.   This practice reduced agitated Veteran behaviors by 14 percent, reduced negative caregiver reactions to behaviors by 34 percent, and reduced caregiver burnout, after one year. |
| 2 | Process Improvement^3^ | Access | Video Blood Pressure Visits (VBPV) | The practice provides virtual visits, allowing Veterans to receive blood pressure check-ins at home, reducing the risk for no-shows and travel expenses for Veterans.  The practice resulted in a 94 percent satisfaction rate among Veterans choosing virtual visits over face-to-face visits, with a 96 percent satisfactory rate. |
| 3 | Clinical Intervention^3^ | Care Coordination | Advanced Comprehensive Diabetes Care (ACDC) | The program is a nurse-administered telemedicine intervention which addresses advanced diabetes, using standard telemonitoring services to deliver diabetes self-management support and clinician-guided medication management.  This practice improved diabetes test results (HbA1c) by an average of 1.0 percent versus usual care over a six-month period, and by nearly 2.0 percent among engaged Veterans. |
| 4 | Clinical Intervention | Care Coordination | Reducing Chronic Obstructive Pulmonary Disease (COPD) Readmission Rates by Improving Transitions of Care through an Interprofessional Primary Care Team | The practice uses an action plan to reduce COPD readmissions including interprofessional visits, referrals for pulmonary rehabilitation, tobacco cessation treatment, and optimized medications.  This practice demonstrated a significant reduction in total number of readmissions, with 0 percent of patients in the program readmitted compared to 18.9 percent for standard of care. |
| 5 | Staff Intervention | Provider Recruitment and Retention in Rural Facilities | Registered Nurse (RN) Stay Interviews: A Nursing Turnover Strategy | The practice uses an interview plan with new nursing staff at regular intervals during their first year to assess satisfaction and boost retention.  This practice resulted in a 65 percent reduction in RN turnover over five quarters and an improvement of 35 positions in national ranking. |
| 6 | Clinical Intervention | Quality and Safety | Better Health, Lower Cost: How IMPaCT Community Health Workers Can Support Veterans | IMPaCT provides training to community health workers (CHWs) to prepare them to join a health care team and provide social support to high risk patients.  This practice reduced total patient hospital days by nearly ten-fold at six months (13 vs. 138) with persistence of effect at nine months (69 vs. 162 days), and patients were twice as likely to report high-quality primary care. |
| 7 | Clinical Intervention | Quality and Safety | Getting Hospitalized Veterans Back on Their Feet: The STRIDE Program | STRIDE is a supervised walking program for hospitalized older adults designed to address immobility during hospitalization and its negative consequences. The program provides hospitalized older Veterans with a gait and balance assessment by a physical therapist followed by supervised walks with a nursing assistant throughout their hospital stay.  This practice resulted in shorter hospital stays for Veterans that participated from 5.7 days to 4.7 days and saved the hospital approximately $1,742 per Veteran that participated in the program. |
| 8 | Process Improvement^3^ | Quality and Safety | Utilizing Machine Learning to Audit and Impact Prescription Benefit Decisions | The practice uses a Deep Learning Algorithm (DLA) to identify and reduce prescription drug billing errors associated with a service-connected condition. |
| 9 | Process Improvement | Veteran Experience | Environmental Management Service (EMS) | The practice provides room cards with QR codes that employees and patients can use to submit concerns or feedback to the EMS team 24 hours a day. This practice improved facility Survey of Healthcare Experiences of Patients (SHEP) by more than 27 percent/ |
| 10 | Clinical Intervention^3^ | Veteran Experience | CharlieFit (Gerofit) – Managing Diseases and Lowering Care Costs with Exercise | The program provides older Veterans with personally tailored exercises. 92 percent of participating Veterans reported high satisfaction with the program and noted that this program often provides them with their “most positive VA experience.” Additionally, the program improved functional status, CV metabolic profile, and reported post-traumatic stress disorder (PTSD) and anxiety symptoms among participating Veterans. |
| **Cohort 5 Promising Practice Descriptions** | | | | |
| **Number** | **Practice Type** | **DoE Category** | **Name** | **Short Description** |
| 1 | Process Improvement^3^ | Access | One Consult Pathway: Whole Health and Pain Management Implementation | It can be quite confusing to orchestrate the many non-opioid services available to reduce pain and improve function. At VA Central Iowa Health Care System, One Consult Pathway helps providers ensure that Veterans receive the appropriate professional and self-care to address not only pain but also their mission, aspirations, and purpose in life. One Consult has resulted in more Veterans receiving the help they desire (dramatic increase in consult volume the first month post-implementation with sustained trajectory in the second and third quarter of Fiscal Year (FY) 2019 by an additional 54%) and primary care providers feeling good about facilitating that help (satisfaction inverted from 80% dissatisfied prior to One Consult to 80% very satisfied post-implementation). |
| 2 | Clinical Intervention^3^ | Digital Health | TeleRehab Wheeled Mobility Clinic to Contract Nursing Home (CNH) | Contract nursing home facilities, serving a large geographical area, manage an increasing population of Veterans with significant complex medical needs and deserve our partnership and clinical expertise in wheeled mobility assessments and delivery. Telerehab wheeled mobility clinic bridges the gap for rural access by eliminating travel for Veterans, increases Veteran satisfaction, encourages vendor partnership, and decreases VA operational cost by using a digital platform to complete telehealth visits. With minimal overhead cost, this program can be implemented within any VA by using current staff and existing equipment to meet the needs of Veterans utilizing community care. |
| 3 | Process Improvement | Quality and Safety | Pre-operative Frailty Screening and Prehabilitation | Frail patients suffer disproportionately high rates of adverse postoperative complications, including death—even after seemingly “minor” surgeries. By implementing rapid, point-of-care frailty assessment in outpatient surgery clinics, it becomes possible to identify high risk patients to improve outcomes through a variety of interventions such as interdisciplinary case review, palliative care consultation to clarify frailty-specific goals of care, and “Prehabilitation” with preoperative strength, endurance, and nutritional training. Initial implementation is associated with a three-fold survival advantage among the frail with as many as 1 in 5 frail patients electing non-operative management with very low decision regret. |
| 4 | Staff Intervention | Quality and Safety | Suicide Alert Red Card | The Suicide Alert Red Card provides frontline personnel a bold color silent signal that is modifiable, easily reproduced, and budget-friendly but mostly a proactive tool to be waved alerting coworkers for help with a suicidal caller. The Suicide Alert Red Card is printed and laminated with contact information for several Mental Health staff members that will assist by coming to the area and taking over the call. The Suicide Alert Red Card has reduced mental health response time of Mental Health to seconds as the suicidal caller has reached out with the last bit of their will to live leaving their fate lying in our hands. |
| 5 | Process Improvement | Quality and Safety | Veterans Mental Evaluation Team (VMET) | This practice involves the proactive utilization of VA Police Department (VAPD) officers in conjunction with mental health clinicians to conduct outreach contacts and follow-up on cases of Veterans experiencing mental health issues/crises. The Veterans Mental Evaluation Team (VMET) coordinates with community police officers to have a VA presence on mental health crisis calls in the community. It reaches high-risk Veterans, both connected and not connected to psychiatric care, and those in mental health crisis. |
| 6 | Clinical Intervention^3^ | Improving Capacity, Efficiency, and Productivity | Tele-Dentistry | This program provides quality dental care to Veterans via Store-and-Forward Technology (SFT) avoiding the need for an in-person appointment. The program was developed to increase access to care and provider productivity, while improving efficiency using minimal resources. |
| 7 | Clinical Intervention | Veteran Experience | Fall Boot Camp for Veterans Suffering from Parkinson's Disease | An eight-week program developed to decrease falls in Veterans with Parkinson’s disease. The program consists of four stations that focus on improved gait through Rhythmic Auditory Stimulation and multitasking while walking; improved balance and coordination through Tai Chi, use of Wii, and balance obstacle course; and improved power and amplitude through boxing. Four participants meet for one hour each week for eight weeks and rotate between four stations for fifteen minutes each with pre- and post-treatment and three-month follow up testing of gait, dynamic balance, lower body power, and cognition |
| 8 | Clinical Intervention | Veteran Experience | Geriatric Emergency Room Innovations for Veterans (GERI-VET) | GERI-VET is the first comprehensive geriatric emergency department (ED) program within the VHA, providing ED personnel with training in multidisciplinary geriatric evaluation and care coordination. GERI-VET provides evidence-based emergency care for aging-specific needs and ensures timely referrals to VA home and community resources |
| 9 | Process Improvement^3^ | Veteran Experience | Virtual Claims Clinic Program | The Virtual Claims Clinic provides an effective and flexible pathway to directly improve the lives of Veterans and their caregivers by using modern video conferencing and software technology. Veterans are provided an opportunity to personally discuss their benefits questions and concerns and submit claims electronically with the Veterans Benefits Administration (VBA) while the Veteran is physically located at a VHA facility. Staff involved in implementation of the program are afforded opportunities to collaborate across health care and administrative disciplines and develop partnerships between agencies, VHA, and VBA. |
| 10 | Clinical Intervention | Whole Health | A Whole Health Approach for Reducing Opioid Reliance and Utilization | This practice improves opioid safety in Veterans on chronic opioids by holding group appointments that introduce concepts of self-care and whole health, safety education, and goal setting. Veterans attend group appointments to introduce self-care modalities such as mindfulness, Yoga, Tai-Chi, and opioid safety education. |
| 11 | Clinical Intervention^3^ | Whole Health | Healthier Kidneys Through Your Kitchen | This practice provides a class explaining Chronic Kidney Disease (CKD) and how to slow its progression. The class includes a Healthy Teaching Kitchen demonstration. Veterans learn about kidney nutrition and are encouraged to schedule nutrition appointments. |
| 12 | Staff Intervention | Workforce Development | Dedicated Environmental Services Training Specialist | This practice develops professional, knowledgeable technicians who are fundamental to infection control efforts and crucial to establishing an environment where safe and therapeutic health care can be provided. Ongoing training and education emphasize and addresses the complexities of environmental care within a health care environment. It also strives to enhance a Veteran-centric employee experience by incorporating strong preceptor and Whole Health Coaching components. |

^1^Two additional practices were designated as Promising Practices but were implemented outside of standard Diffusion of Excellence processes. These two practices are not included in this evaluation.

- Audiology and Optometry Direct Scheduling (Cohort 1): This practice allows for HAS (Health Administration Services) to schedule routine appointments in Audiology and Optometry without first scheduling a Primary Care appointment to obtain a referral.
- 4-Sight (Cohort 4): This practice is an IT and business solution for the VA Prosthetic and Sensory Aids Service (PSAS) that promotes standardization of procurement of eyeglasses for Veterans by using data to perform automation actions in VistA.

^2^Practices include the following: 1) Clinical Intervention - An intervention that focuses on doing something to/for patients. 2) Staff Intervention - An intervention that focuses on doing something to/for staff. 3) Process Improvement Intervention - An intervention that focuses on redesign of existing processes

^3^Denotes a virtual practice and/or a practice adapted to have virtual components


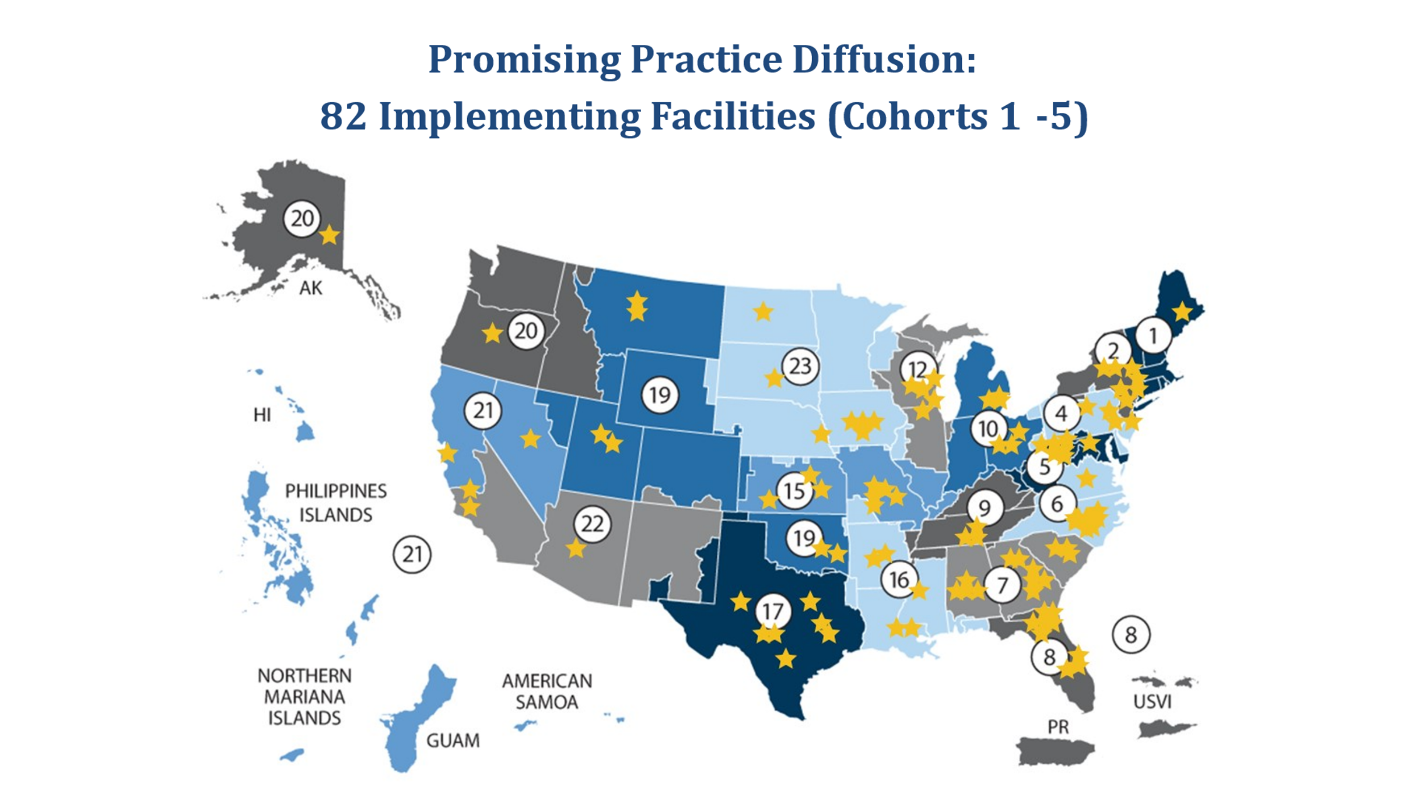


Note: This map illustrates the spread of the 82 Promising Practices across all 21 VHA Veterans Integrated Service Networks during Cohorts 1-5.


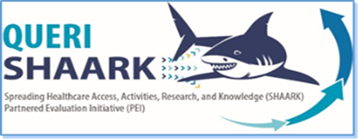
RK PEI Evaluation

## QUERI SHAARK PEI

The SHAARK PEI is funded jointly by the VHA [Quality Enhancement Research Initiative](https://www.queri.research.va.gov) (QUERI) (grant PEC-17-002; 1I50HX002451-01) and VHA [Office or Rural Health](https://www.ruralhealth.va.gov/) in coordination with the Diffusion of Excellence (DoE) program. The [SHAARK PEI](https://www.durham.hsrd.research.va.gov/SHAARK.asp) team formed in 2016 to evaluate the impact of DoE as a program. The evaluation team has been embedded with DoE leadership for most of the history of the DoE program.

Evaluation team members are employees of VHA [Health Services Research & Development](https://www.hsrd.research.va.gov/) (HSR&D) Centers of Innovation (COINs) in [Durham, North Carolina](https://www.durham.hsrd.research.va.gov/) (George L. Jackson, Corresponding Principal Investigator); [Ann Arbor, Michigan](http://www.annarbor.hsrd.research.va.gov/) (Andrea L. Nevedal, Multiple Principal Investigator), and [Bedford/Boston, Massachusetts](http://www.choir.research.va.gov/) (Gemmae M. Fix, Multiple Principal Investigator). For more information on the SHAARK PEI, you can email the Project Coordinator for the corresponding evaluation site, Brandolyn White, at [Brandolyn.White@va.gov](mailto:Brandolyn.White@va.gov).

The SHAARK PEI is an embedded, mixed methods evaluation of DoE [1]. It has been informed by implementation science theories and frameworks, including the Consolidated Framework for Implementation Research [2], Theory of Organizational Readiness for Change [3], and Rogers Diffusion of Innovations [4]. The goal is to provide rapid and frequent feedback to DoE partners about:

- Factors associated with submitting a practice to Shark Tank
- VHA leadership decision-making processes when choosing to bid resources for implementation of a practice
- Influences on implementation success within implementing facilities
- Pathways toward broader diffusion of practices
- Impact of DoE as a model of diffusion

Per regulations outlined in VHA Program Guide 1200.21, this evaluation has been designated a non-research quality improvement activity.

## Bibliography

[1] Jackson, G.L.; Cutrona, S.L.; White, B.S.; Reardon, C.M.; Orvek, E.; Nevedal, A.L.; Lindquist, J.; Gifford, A.L.; White, L.; King, H.A.; DeLaughter, K.; Houston, T.K.; Henderson, B.; Vega, R.; Kilbourne, A.M.; Damschroder, L.J. Merging Implementation Practice and Science to Scale Up Promising Practices: The Veterans Health Administration (VHA) Diffusion of Excellence (DoE) Program. The Joint Commission Journal on Quality and Patient Safety 2021;47:217–27. https://doi.org/10.1016/j.jcjq.2020.11.014.

[2] Damschroder LJ, Aron DC, Keith RE, Kirsh SR, Alexander JA, Lowery JC. Fostering implementation of health services research findings into practice: a consolidated framework for advancing implementation science. Implement Sci 2009;4:50. https://doi.org/10.1186/1748-5908-4-50.

[3] Weiner BJ. A theory of organizational readiness for change. Implementation Sci 2009;4:67. https://doi.org/10.1186/1748-5908-4-67.

[4] Rogers E. Diffusion of innovations: 5th ed. New York: Free Press; 2003.
