# Supplementary material for: Understanding pathways from implementation to sustainment: a longitudinal, mixed methods analysis of promising practices implemented in the Veterans Health Administration
Source: Implement Sci. 2024 May 7;19:34. doi: 10.1186/s13012-024-01361-z (PMC11075255; doi:10.1186/s13012-024-01361-z)
Supplement: Supplementary file 2 — Additional file 2. Full Sustainment Survey. [file 13012_2024_1361_MOESM2_ESM.docx]

# Additional File 2. 2021 Sustainment Survey

2021 DoE Sustainment Survey

We are interested in learning the current status of your practice as well as the impact of the COVID-19 pandemic. Please complete the survey below.

Thank you!

What is the current status of your practice? Discontinued permanently Temporarily not in use/not in place Partially in use/in place

In use/in place


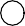

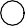

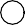

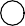


Why is your practice [currentstatus]?

Is this practice considered routine, usual practice? Yes

(i.e., practice is nearly always used or done when Partially


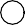

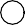

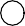


appropriate by all individuals involved) No

Was this practice considered routine, usual practice Yes

when it was in use/in place? (i.e., practice was Partially


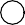

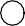

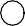


nearly always used or done when appropriate by all No individuals involved)

Please explain:

**Indicate your agreement with the statements below on a scale of 1 to 5:**

This practice is in line with the mission and values of the facility

This practice fits into existing processes and workflows at the facility

1. - N/A: This

item is not relevant to your practice


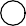


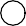


1. - Strongly

Disagree


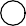


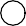


1. - Disagree 3 - Neutral 4 - Agree 5 - Strongly

Agree


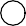

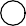

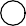

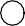


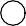

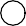

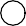

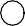


This practice has support from
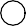

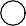

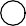

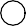

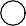

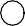
 relevant leadership at the facility

This practice has a Champion
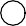

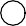

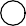

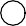

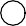

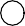
 (leader) at the facility

This practice has sufficient
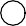

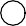

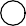

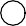

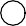

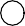
 funding at the facility

This practice has sufficient
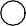

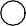

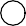

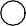

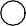

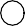
 staffing at the facility

| This practice has sufficient resources (e.g., space, equipment) at the facility | 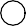 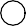 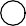 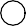 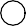 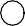  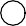 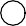 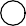 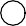 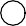 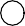  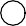 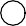 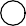 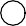 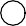 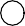 | | | | | |
| --- | --- | --- | --- | --- | --- | --- |
| This practice has priority at the facility |  |  |  |  |  |  |
| This practice has support and buy-in from communities outside the facility |  |  |  |  |  |  |
|  | 0 - N/A: This | 1 - Strongly | 2 - Disagree | 3 - Neutral | 4 - Agree | 5 - Strongly |
|  | item was not | Disagree |  |  |  | Agree |
|  | relevant to | 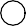 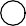 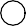 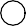 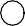  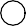 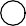 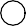 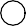 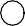  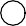 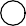 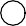 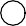 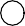  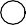 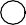 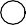 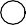 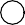  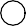 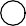 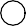 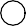 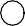  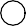 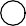 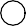 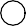 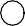  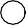 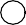 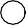 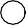 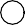  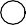 | | | | |
|  | your practice |  |  |  |  |  |
| This practice was in line with the mission and values of the facility |  |  |  |  |  |  |
| This practice fit into existing processes and workflows at the facility |  |  |  |  |  |  |
| This practice had support from relevant leadership at the facility |  |  |  |  |  |  |
| This practice had a Champion (leader) at the facility |  |  |  |  |  |  |
| This practice had sufficient funding at the facility |  |  |  |  |  |  |
| This practice had sufficient staffing at the facility |  |  |  |  |  |  |
| This practice had sufficient resources (e.g., space, equipment) at the facility |  |  |  |  |  |  |
| This practice had priority at the facility |  |  |  |  |  |  |
| This practice had support and buy-in from communities outside the facility |  |  |  |  |  |  |

For items you rated 1 (strongly disagree) or 2 (disagree) are these items barriers to keeping your

practice in use/in place? Please explain.

For items you rated 4 (agree) or 5 (strongly agree) are these items facilitators to keeping your

practice in use/in place? Please explain.

For items you rated 1 (strongly disagree) or 2 (disagree), were these items barriers to keeping

your practice in use/in place? Please explain.

For items you rated 4 (agree) or 5 (strongly agree) were these items facilitators to keeping your

practice in use/in place? Please explain.

Is this practice demonstrating effectiveness at the Yes

facility? Partially

No

Was this practice demonstrating effectiveness at the Yes

facility when it was in use/in place? Partially No

What indication do you use to determine its effectiveness? Please explain:

What indication did you use to determine its effectiveness when it was in use/in place? Please explain:

Have there been any changes or adaptations to this Yes

practice? No

Unsure

Were there any changes or adaptations to this Yes

practice at the facility when it was in use/in No

place? Unsure

Why?

Why not?

Please explain:

What is the likelihood this practice will be in Very Unlikely

use/in place at the facility in the future? Unlikely

Neither Unlikely nor Likely Likely

Very Likely

Please explain what will make keeping your practice in use/in place harder at the facility:

Please explain what will make keeping your practice in use/in place easier at the facility:

What impact did the COVID-19 pandemic have on this practice at the facility?
