# Supplementary material for: Understanding pathways from implementation to sustainment: a longitudinal, mixed methods analysis of promising practices implemented in the Veterans Health Administration
Source: Implement Sci. 2024 May 7;19:34. doi: 10.1186/s13012-024-01361-z (PMC11075255; doi:10.1186/s13012-024-01361-z)
Supplement: Supplementary file 3 — Additional file 3. Sustainment outcome by practice type. Sustainment outcome by presence/absence of a virtual component. Sustainment outcome by sustainability outcome. [file 13012_2024_1361_MOESM3_ESM.docx]

# Additional File 3

## 2021 Sustainment Outcome by Practice Type

| **Sustainment Outcome** | **Clinical Interventions** | **Process**  **Improvements** | **Staff**  **Interventions** | **Total** |
| --- | --- | --- | --- | --- |
|  | Focuses on doing something to/for patients | Focuses on redesign of existing processes | Focuses on doing something to/for staff |  |
| Fully sustained | 13 (45%) | 13 (45%) | 3 (10%) | 29 |
| Not fully sustained | 15 (50%) | 13 (43%) | 2 (7%) | 30 |
| Missing | 6 (26%) | 9 (39%) | 8 (35%) | 23 |
| Total | 34 (41%) | 35 (43%) | 13 (16%) | 82 |

## 2021 Sustainment Outcome by Virtual Practices

| **Sustainment Outcome** | **Virtual component** | **No Virtual component** | **Total** |
| --- | --- | --- | --- |
| Fully sustained | 13 (45%) | 16 (55%) | 29 |
| Not fully sustained | 11 (37%) | 19 (63%) | 30 |
| Missing | 7 (30%) | 16 (70%) | 23 |
| Total | 31 (38%) | 51 (62%) | 82 |

## 2021 Sustainment Outcomes by Sustainability Status

| **Sustainment outcome** | **Sustainability Outcome:  “Very Likely”** | **Sustainability Outcome:  “Likely”** | **Sustainability Outcome:  “Neither Likely nor Unlikely”** | **Sustainability Outcome:  “Unlikely”** | **Sustainability Outcome:  “Very Unlikely”** | **Missing** | **Total** |
| --- | --- | --- | --- | --- | --- | --- | --- |
| Sustained | 23 (79%) | 4 (14%) | - | - | 1 (3%) | 1 (3%) | 29 |
| Partially in use/in place | - | 2 (33%) | - | 2 (33%) | 2 (33%) | - | 6 |
| Temporarily not in use/in place | 2 (22%) | 3 (33%) | 3 (33%) | - | 1 (11%) | - | 9 |
| Total^1^ | 25 (57%) | 9 (20%) | 3 (7%) | 2 (5%) | 4 (9%) | 1 (2%) | 44 |

^1^44 representatives received the sustainability outcome question. The remaining representatives had discontinued their practice (15) or did not respond to the entire survey (23).
